# Identification and Pathogenicity of Pestalotioid Species on *Alpinia oxyphylla* in Hainan Province, China

**DOI:** 10.3390/jof10060371

**Published:** 2024-05-22

**Authors:** Xiufen Cui, Zhigang Hao, Menghuai Chen, Shuang Song, Jinan Zhang, Yingbin Li, Jianqiang Li, Yixiang Liu, Laixin Luo

**Affiliations:** 1 Beijing Key Laboratory of Seed Disease Testing and Control, Department of Plant Pathology, College of Plant Protection, China Agricultural University, Beijing 100193, China; cuixiufencoco@163.com (X.C.); 17810266056@163.com (Z.H.); menghuaic@163.com (M.C.); songshuang0611@163.com (S.S.); zjn975393389@163.com (J.Z.); lijq231@cau.edu.cn (J.L.); 2 Key Laboratory of Surveillance and Management for Plant Quarantine Pests, Ministry of Agriculture and Rural Affairs, China Agricultural University, Beijing 100193, China; 3 Department of Pesticide Science, College of Plant Protection, Yunnan Agricultural University, Kunming 650201, China; liyb30@163.com; 4 Department of Plant Pathology, College of Plant Protection, Yunnan Agricultural University, Kunming 650201, China

**Keywords:** pestalotioid species, new taxa, *Alpinia oxyphylla*, ring leaf blight, pathogenicity

## Abstract

*Alpinia oxyphylla* is a traditional Chinese medicinal plant with a medicinal history of more than 1700 years. Ring leaf blight (RLB) disease, caused by pestalotioid species, is an important disease of *A. oxyphylla*, seriously affecting the yield and quality of its fruits. The causal agent of RLB disease has not been systematically identified or characterized yet. In this study, thirty-six pestalotioid strains were isolated from the leaves and stems of *A. oxyphylla* that was collected from six cities of Hainan province, China. Based on the multi-locus phylogeny (*ITS*, *tef-1α* and *tub2*) and morphological characteristic analyses, seventeen species belonging to three genera (*Neopestalotiopsis*, *Pestalotiopsis* and *Pseudopestalotiopsis*) were identified, and six new species (*N. baotingensis*, *N. oblatespora*, *N. olivaceous*, *N. oxyphylla*, *N. wuzhishanensis* and *N. yongxunensis*) were described. Pathogenicity tests revealed that strains of *Neopestalotiopsis* species caused more severe ring leaf blight on *A. oxyphylla* than strains of *Pestalotiopsis* and *Pseudopestalotiopsis* under wounded inoculation conditions.

## 1. Introduction

*Alpinia oxyphylla* belongs to the family Zingiberaceae and is an important Chinese herbal plant, with a medicinal history dating back 1700 years [1]. As an edible herb, the traditional medicinal effects of *A. oxyphylla*’*s* fruit mainly include warming the kidney, stopping spermatorrhea, arresting polyuria, warming the spleen as well as stopping diarrhea and excess saliva [2,3]. Moreover, the essential oil of *A. oxyphylla* has various effects including antibacterial, anticancer, antioxidant, vasodilation and improved immunity [4]. *A. oxyphylla* likes to grow in warm and humid environmental conditions and is commonly planted under rubber trees, areca trees and other economic forests as a semi-shade plant [5,6,7]. *A. oxyphylla* is mainly distributed in southern China, such as in Hainan, Guangdong and Guangxi provinces. Among them, Hainan, with abundant rainfall and high temperatures, is the most important planting area for *A. oxyphylla*, accounting for 90% of the total output in China [8,9,10].

The occurrence of diseases causes serious losses to the production and quality of *A. oxyphylla*. Ring leaf blight (RLB) is an important disease of *A. oxyphylla* that occurs from the seedling to the fruiting stage, mainly infecting old leaves. The disease often extends from the leaf edge or tip, forming irregular, reddish-brown spots with alternating dark and light brown wavy concentric rings and obvious yellow halos around the periphery of the disease spots, on which numerous small black conidiomata of the pathogen are scattered. The pathogen of this disease can be transmitted through wind and rain, mainly invading through wounds. The high temperature and rainy season contribute to the occurrence of RLB disease, and the high incidence of this disease is from August to September. Under suitable conditions, the proportion of diseased plants can reach more than 50%, and the area of the diseased spots can reach 1/3–1/2 of the leaf surface, even the entire leaf, which has an impressive impact on the growth of *A. oxyphylla* [11,12].

The pathogen of RLB disease was first reported as *Pestalotia palmarum* in 1986 [11]. Subsequently, the classification status of *P. palmarum* was adjusted to the genus *Pestalotiopsis*, while *Pestalotia* and *Pestalotiopsis* were used confusingly in descriptions of *A. oxyphylla* diseases [13]. The ring brown spot (RBS) disease of *A. oxyphylla* was caused by *Pestalosphaeria alpinia*, a sexual morph of pestalotioid fungi [14]. As asexual fungi, most pestalotioid species lack the sexual morphs *Pestalosphaeria* [15]. Most of pestalotioid species are important plant pathogens and are also commonly found as endophytes or saprophytes, being mainly distributed throughout tropical and temperate regions [16,17,18]. Pestalotioid species can infect the leaves, shoots, flowers, fruits or other parts of plants and cause a variety of diseases in multiple economic crops, including leaf spots, gray blight, shoot dieback, trunk diseases, dry flowers and fruit rot [17,19,20,21,22,23,24,25,26]. Hence, pestalotioid species causing disease in *A. oxyphylla* need to be reidentified and characterized based on their fungal diversity, molecular systematics and pathogenicity.

The development of a molecular phylogenetic analysis overcomes the limitation of overlapping conidial measurements in the traditional taxonomy of pestalotioid species [16,17,27,28]. In 2014, two novel genera, *Neopestalotiopsis* and *Pseudopestalotiopsis*, were segregated from *Pestalotiopsis* based on conidial characters and multi-locus phylogenetic analyses. The combined sequences of the *ITS*, *tub2* and *tef-1α* genes were used to construct phylogenetic trees, which become an important basis for distinguishing different species within the genera *Pestalotiopsis*, *Neopestalotiopsis* and *Pseudopestalotiopsis*. Morphologically, *Neopestalotiopsis* can be easily differentiated from *Pestalotiopsis* and *Pseudopestalotiopsis* by the versicolorous median cells of the conidia, and *Pseudopestalotiopsis* is different from *Pestalotiopsis* with its three darker, concolorous median cells [17]. Through these methods, many novel pestalotioid species isolated from different plants have been introduced in recent years [19,22,29,30,31,32,33,34].

Therefore, the objective of this study is to clarify the types, characteristics and pathogenicities of pestalotioid species related to disease in *A. oxyphylla* of Hainan, China.

## 2. Materials and Methods

### 2.1. Sample Collection, Fungi Isolation and Morphological Examination

Fresh leaves of *A. oxyphylla* with typical ring spots and stems with irregular cloud-like spots were collected from the main planted areas at ten townships in six cities of Hainan province, including Baoting, Ledong, Qiongzhong, Sanya, Wanning and Wuzhishan in 2022. Small pieces (5 mm × 5 mm) of leaves or stems were cut from the junctions of diseased and healthy areas, disinfected with 3% sodium hypochlorite for 3 min, then 75% ethanol for 30 s and subsequently washed with sterilized water three times. The treated tissue pieces were dried on sterilized blotting paper and then placed on PDA plates (containing 100 μg/mL streptomycin, 50 μg/mL kanamycin and 100 μg/mL ampicillin). The plates were cultured at room temperature and examined daily for 7 days; then, the marginal mycelia with different morphologies on each plate were transferred to fresh PDA; subsequently, the pestalotioid strains were purified using single-spore culturing according to the results of the *ITS* sequence analysis.

The pestalotioid strains usually sporulated at room temperature on PDA after 10–20 days. The conidiomata were observed using a dissecting microscope (CNOPTEC, SZ680, Chongqing, China), and the characteristics of spores and conidiophores were observed using an optical microscope (CNOPTEC, DV320, Chongqing, China). All the morphological characteristics of the spores were photographed and measured for at least 30 individuals using OPTPro v.6.1.1.67. The images were processed using Adobe Photoshop CS6. The pure cultures of isolated fungal strains were stored in the seed health center of China Agricultural University.

### 2.2. DNA Extraction, Gene Sequencing and Phylogenetic Analyses

DNA was extracted from fresh fungal mycelia using the Biomed genomic DNA extraction kit (Biomed, Beijing, China). The partial sequences of three genes (*ITS*, *tef-1α* and *tub2*) were amplified. The PCR was performed according to Table 1, and the PCR products were purified and sequenced at Beijing Tsingke Biotech (Beijing, China).

The nucleotide sequences were checked using Chromas2.4.1 and then analyzed using the BLAST tool on the NCBI platform to assess the closest phylogenetic matches. All related sequences determined using BLAST or referenced previous studies were downloaded from GenBank (Table 2). MAFFT v.7 (https://mafft.cbrc.jp/alignment/software/, accessed on 15 October 2023) was used to align each locus sequence, and MEGA v.11 was used to manually improve the sequences. The three final aligned gene sequences were concatenated using SequenceMatrix (13 May 2024) [40].

The phylogenetic analyses of the combined sequences were carried out using maximum-likelihood (ML) and Bayesian inference (BI) methods. The ML analysis was performed on the CIPRES web portal (https://www.phylo.org, accessed on 31 October 2023) using RAxML-HPC BlackBox 8.2.10, with a GTRGAMMA substitution model and 1000 bootstrap replicates [91]. The BI analysis was implemented using MrBayes v.3.2.7 [92], and MrModeltest 2.2 [92] was used to seek the best-fit nucleotide substitution models for each gene. Two Markov chain Monte Carlo (MCMC) methods were run for 1,000,000 generations, and trees were sampled every 1000th generation. The first 25% of trees, standing for the burn-in phase of the analyses, were discarded, and the remaining trees were estimated to be the posterior probabilities. The ML tree and BI tree were viewed using Figtree v.1.4.4. and modified using WPS Office v.12.1.0.16729.

The new species can be further confirmed through PHI (Pairwise Homoplasy Index) analysis, which can also be used to analyze the species’ boundaries and related taxa [93]. The PHI test was completed using SplitsTree v.4 [94,95], and a value over 0.05 revealed no significant recombination in the dataset. The relationships among closely related species were shown using splits graphs through the LogDet transformation and split decomposition.

### 2.3. Pathogenicity Test

The pathogenicity of the fungi was tested using the wound inoculation method. Fresh and healthy leaves of *A. oxyphylla* measuring 30–40 cm long were collected from the field. The surface of the leaves was disinfected by spraying them with 75% ethanol and then washed three times with sterile water. Each fungal isolate was inoculated on 6 sites per leaf with 3 leaf replicates. A piece of mycelium (6 mm diameter), which was taken from the margin of a fresh colony cultured to 2/3 of the PDA plate’s diameter, was placed on the wound of injured leaf using a sterilized needle. A piece of PDA without mycelium was used as the control. The inoculated leaves were placed in a box and cultured in the incubator at 26 °C and 600 LUX, with a 16 h/8 h LED light/dark cycle. After 5 days, disease symptoms were recorded, the lesion area was measured using ImageJ v.1.53c and the data were analyzed using SPSS Statistics 24. The re-isolated fungi from the disease lesion were identified and tested using Koch’s postulates.

## 3. Results

### 3.1. Phylogenetic Analyses

A total of 36 pestalotioid isolates were obtained from the leaves (32 isolates) and stems (4 isolates) of *A. oxyphylla* from six cities in Hainan province. Based on the *ITS* sequence and color of the intermediate cells of the conidia, 36 strains were classified into three genera, of which 32 strains belong to *Neopestalotiopsis*, 2 strains belong to *Pestalotiopsis* and 2 strains belong to *Pseudopestalotiopsis*.

**Figure 1:**
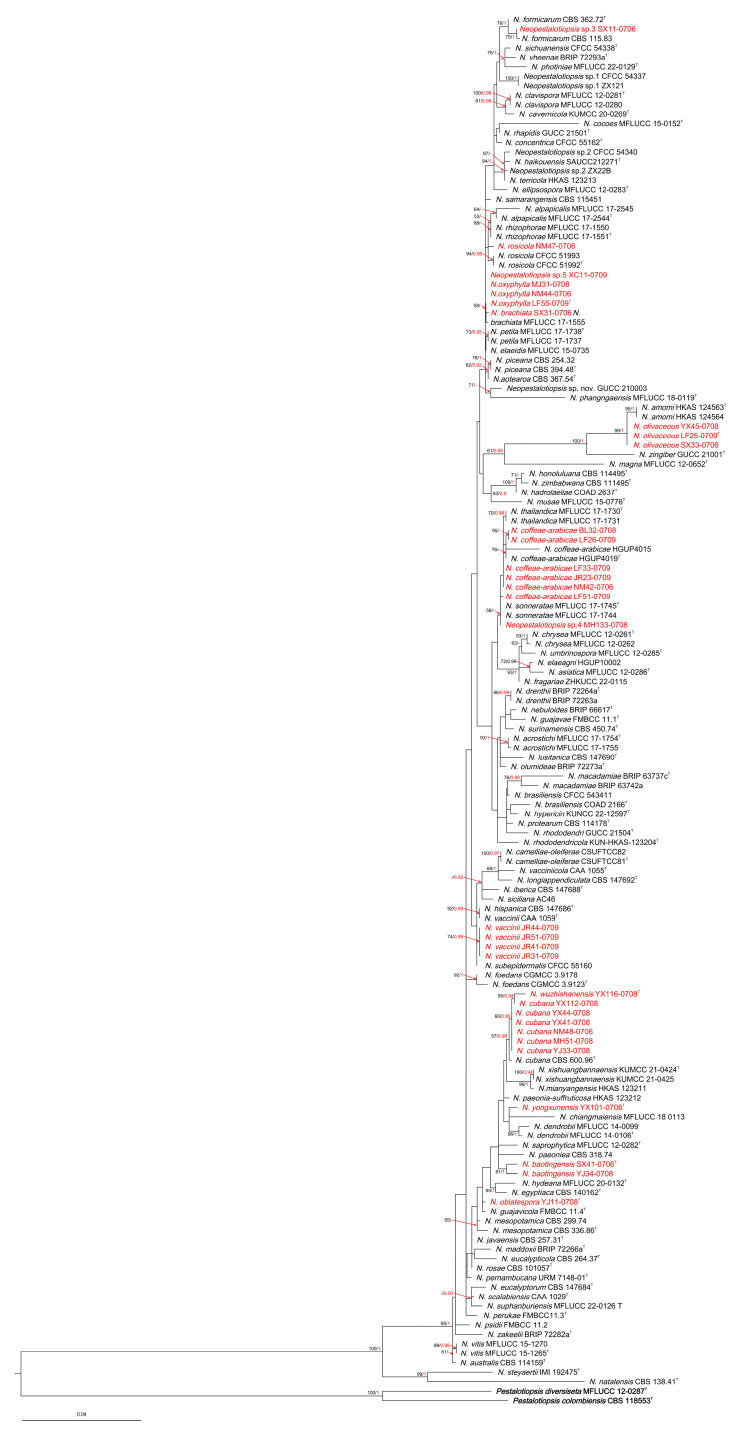
RAxML tree of *Neopestalotiopsis* isolates based on *ITS*, *tef-1α* and *tub2* sequences. The roots of this tree are *Pestalotiopsis diversiseta* MFLUCC 12-0287 and *P. colombiensis* CBS 118553. The strains isolated in this study are marked in red. Ex-type strains are marked with ^T^. ML bootstrap values ≥ 50% and BI probabilities (in red) ≥ 0.90 are displayed at the nodes.

The phylogenetic tree of *Neopestalotiopsis* contained 145 taxa, with 2 outgroup taxa (*Pestalotiopsis colombiensis* and *P. diversiseta*). A total of 1404 characters, including gaps (503 for *ITS*, 469 for *tef-1a* and 432 for *tub2*), were included in the phylogenetic analysis. For the Bayesian inference, the HKY + G model with a gamma-distributed rate was selected for *ITS*, the HKY + G model with a gamma-distributed rate was selected for *tef1-a* and the HKY + I + G model with an invgamma-distributed rate was selected for *tub2*. Similar tree topologies were acquired using the ML and BI methods, and the best scoring ML tree is shown in Figure 1. The phylogenetic tree depicts 32 *Neopestalotiopsis* taxa isolated from *A. oxyphylla*, revealing 6 novel species.

**Figure 2:**
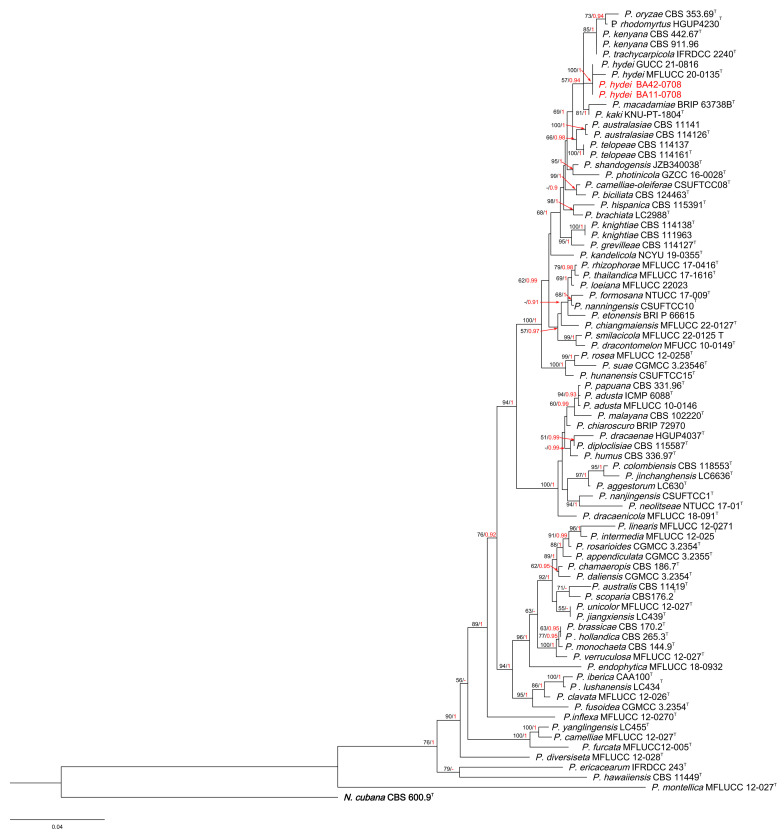
RAxML tree of *Pestalotiopsis* isolates based on *ITS*, *tef-1α* and *tub2* sequences. The root of this tree is *N. cubana* CBS 600.9. The strains isolated in this study are marked in red. Ex-type strains are marked with ^T^. ML bootstrap values ≥ 50% and BI probabilities (in red) ≥ 0.90 are displayed at the nodes.

The phylogenetic tree of *Pestalotiopsis* comprised 78 taxa, with the outgroup taxon *N. cubana* CBS 600.96. A total of 1457 characters, including gaps (505 for *ITS*, 495 for *tef-1a* and 457 for *tub2*), were included in the phylogenetic analysis. For the Bayesian inference, the GTR + I + G model with an invgamma-distributed rate was selected for *ITS*, the GTR + G model with a gamma-distributed rate was selected for *tef1-a* and the GTR + I + G model with an invgamma-distributed rate was selected for *tub2*. Similar tree topologies were obtained using the ML and BI methods, and the best scoring ML tree is shown in Figure 2. The phylogenetic tree depicts two *Pestalotiopsis* strains isolated from *A. oxyphylla*, clustered with the type species of *P. hydei*.

**Figure 3:**
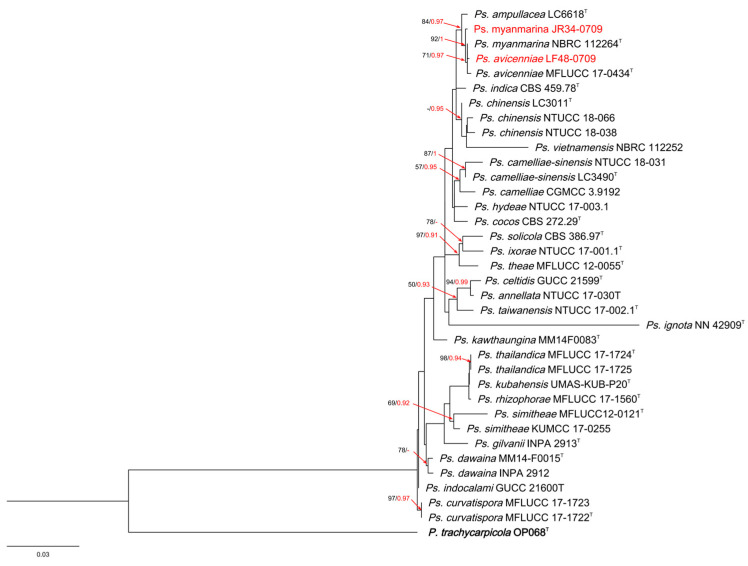
RAxML tree of *Pseudopestalotiopsis* isolates based on *ITS*, *tef-1α* and *tub2* sequences. The root of this tree is *P. trachycaroicola* OP068. The strains isolated in this study are marked in red. Ex-type strains are marked with ^T^. ML bootstrap values ≥ 50% and BI probabilities (in red) ≥ 0.90 are displayed at the nodes.

The alignment of *Pseudopestalotiopsis* contained 35 taxa, with *P. trachicarpicola* OP068 as the outgroup taxon. A total of 1392 characters, including gaps (521 for *ITS*, 442 for *tef-1a* and 429 for *tub2*), were included in the phylogenetic analysis. For the Bayesian inference, the HKY + G model with a gamma-distributed rate was selected for *ITS*, the HKY + G model with a gamma-distributed rate was selected for *tef1-a* and the HKY + I model with a propinv-distributed rate was selected for *tub2*. Similar tree topologies were obtained using the ML and BI methods, and the best scoring ML tree is shown in Figure 3. The phylogenetic tree depicts two *Pseudopestalotiopsis* taxa isolated from *A. oxyphylla*, clustered with the type species of *Ps. avicenniae* and *Ps. myanmarina*, respectively.

### 3.2. PHI Analyses

**Figure 4:**
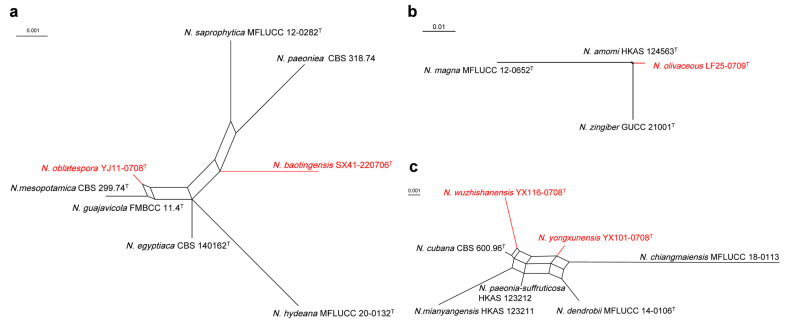
Split graphs showing the results of the PHI test of new (**a**) *N. baotingensis* SX41-0706, *N. oblatespora* YJ11-0708, (**b**) *N. olivaceous* LF25-0709, and (**c**) *N. yongxunensis* YX101-0708 and *N. wuzhishanensis* YX116-0708 with their most closely related species. The new species in each graph is shown in red font. Ex-type strains are marked with “^T^”.

The results of the PHI test indicate no obvious recombination (Φw = 0.1064) among *N. baotingensis* SX41-0706, *N. oblatespora* YJ11-0708 and their closely related species *N. saprophytica* MFLUCC 12-0282, *N. paeoniea* CBS 318.74, *N. hydeana* MFLUCC 20-0132, *N. egyptiaca* CBS 140162, *N. guajavicola* FMBCC 11.4 and *N. mesopotamica* CBS 299.74 (Figure 4a). And there is no significant recombination (Φw = 0.0786) between *N. olivaceous* LF25-0709 and its closely related species *N. amomi* HKAS 124563, *N. zingiberis* GUCC 21001 and *N. magna* MFLUCC 12-0652 (Figure 4b). *N. yongxunensis* YX101-0708, *N. wuzhishanensis* YX116-0708 and their closely taxa have no significant recombination according to the PHI test results (Φw = 0.1103) (Figure 4c).

### 3.3. Taxonomy

Based on the multi-locus phylogeny (*ITS*, *tef-1α* and *tub2*) and morphological characteristic analyses, 17 species were identified. Three *Neopestalotiopsis* strains failed to acquire spores and were not identified as specific species. Six new species are described below. The conidial dimensions of the identified isolates in this study and their closely related strains are shown in Table 3.

**Figure 5:**
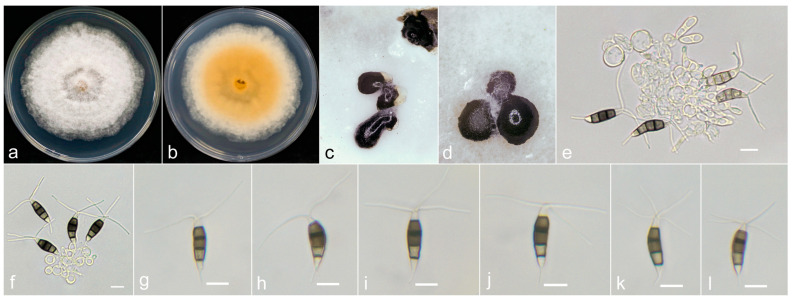
*Neopestalotiopsis baotingensis* (SX41-0706, holotype). (**a**,**b**) Colony on PDA (above and reverse), (**c**,**d**) conidiomata on PDA, (**e**,**f**) conidiogenous cells and (**g**–**l**) conidia. Scale bars = 10 μm.

***Neopestalotiopsis baotingensis*** X.F. Cui and Z.G. Hao, sp. nov. (Figure 5).

MycoBank: MB854050.

**Etymology:** It is named in reference to the first collection city of Baoting in Hainan Province.

**Holotype:** SX41-0706.

**Description:**

The conidiomata on PDA are solitary or aggregated, globose and dark. The conidiophores often degenerated to conidiogenous cells. Conidiogenous cells are spherical and hyaline. The conidia are fusiform, straight to slightly curved, 18–26 × 5–7.2 μm ($\bar{x}$ = 23.2 × 6.3 μm) and have four septa. The basal cell is conical to obtuse, hyaline, thin and smooth walled and is 3.2–6.2 μm long ($\bar{x}$ = 4.5 μm). The three median cells are 12–17.3 μm ($\bar{x}$ = 14.8 μm), verruculose, versicolored, pale brown to dark brown and the septa and periclinal walls are darker than the rest of the cell. The second cell from the base is pale brown to brown, is paler than the two other cells and is 3.2–5.5 μm long ($\bar{x}$ = 4.4 μm). The third cell is brown to dark brown, darker than the two other cells and is 4–6 μm long ($\bar{x}$ = 4.9 μm). The fourth cell is brown to dark brown and 4–6 μm long ($\bar{x}$ = 5 μm). The apical cell is 2.5–5 μm long ($\bar{x}$ = 3.8 μm) and cylindric to subcylindric, with 2–4 tubular appendages on it, often 2–3, arising from its apex, which are unbranched and 3–30.5 μm long ($\bar{x}$ = 19.7 μm). The single basal appendage is unbranched, tubular, centric and 2.5–10 μm long ($\bar{x}$ = 6.3 μm). A sexual morph was not observed.

**Culture characteristics**: The colony reached 70 mm in diameter on PDA after 4 days of growth at room temperature. The colony was off white, with dense aerial hyphae on the surface with crenate edges, and its reverse was lemon yellow.

**Material examined**: The sample originated in China, Hainan Province, Baoting city, Shiling Township, Shuixian village, from leaf spots of *A. oxyphylla*, which was collected on 6 July 2022 by X.F. Cui and Z.G. Hao (SX41-0706, holotype); the ex-type came from Hainan Province, Wuzhishan city, Shuiman Township, Yongxun village, from spots on the stem base of *A. oxyphylla*, which was collected on 8 July 2022 by X.F. Cui and Z.G. Hao (YJ34-0708);

**Notes**: Two strains of *Neopestalotiopsis baotingensis* were isolated from two cities in Hainan, SX41-0706 and YJ34-0708, with well-supported clusters (ML = 81%, BI = 1). *N. baotingensis* is closely related to *N. saprophytica* (MFLUCC 12-0282) in the phylogenetic analysis. The conidiophores of *N. baotingensis* often degenerated to conidiogenous cells, while those of *N. saprophytica* were unbranched or irregularly branched; *N. baotingensis* is shorter than *N. saprophytica* (*N. baotingensis* 18–26 μm, $\bar{x}$ = 23.2 μm vs. *N. saprophytica* 22–30 μm, $\bar{x}$ = 24.9 μm); *N. baotingensis* has shorter apical appendages (*N. baotingensis* 3–30.5 μm, $\bar{x}$ = 19.7 μm vs. *N. saprophytica* 23–35 μm, $\bar{x}$ = 27.3 μm). Additionally, there was an 21 bp difference for *ITS*~*tef-1α*~*tub2* between *N. baotingensis* and *N. saprophytica* (4/452 in *ITS*; 16/746 in *tef-1α* and 1/415 in *tub2*). The PHI test for *N. baotingensis* revealed that there is no obvious recombination between *N. baotingensis* and its closely related taxa. Therefore, *N. baotingensis* is classified as a new species in this study.

**Table 3:** The conidial dimensions of pestalotioid species related to this study.

Species	Isolate Number	Conidial Size (μm)	Apical Appendages (μm)		Basal Appendages
			Number	Length	
***N. baotingensis***	SX41-0706	18–26 × 5–7.2	2–4	3–30.5	2.5–10
*N. saprophytica*	MFLUCC 12-0282	22–30 × 5–6	2–4	23–35	4–7
***N. brachiata***	SX31-0706	18.5–25.3 × 5.5–7.5	1–3	3.7–38.7	2.5–8
*N. brachiata*	MFLUCC 17-1555	18.5–25 × 5.5–6	1–3	9.5–33	4–9
***N. coffeae-arabicae***	BL32-0708	19.2–25.3 × 5.3–7	2–4	10.9–22.6	1.4–5.4
***N. coffeae-arabicae***	LF51-0709	17.8–24.2 × 5–7	2–4	6.6–21.6	2.5–6.8
***N. coffeae-arabicae***	NM42-0706	17.5–23.8 × 5.8–7.8	2–4	12.7–31	2.7–9.2
*N. coffeae-arabicae*	HGUP4019	16–20 × 5–7	2–4	11–16	3–5
***N. cubana***	MH51-0708	19.7–30 × 5–6.8	2–4	15.5–32.2	4–7.5
***N. cubana***	YX112-0708	21–29 × 5.6–7.3	2–4	18.7–36.5	3.3–10.3
*N. cubana*	CBS 600.96	20–25 × 8–9.5	2–4	21–27	4–7
***N. oblatespora***	YJ11-0708	18–23.2 × 5.5–6.7	2–4	10–26.5	2–9
*N. guajavicola*	FMBCC 11.4	23.3 × 6.5	2–3	21.8	4.4
***N. olivaceous***	LF25-0709	21.5–33.8 × 5.5–7.7	2–5	9.5–22.5	(0) 1.2–4.8
*N. amomi*	HKAS 124563	18–30 × 4–7	2–3	7–17	2–5
*N. zingiberis*	GUCC 21001	21–31 × 6–9.5	1–3	12–15	0–6
***N. oxyphylla***	LF55-0709	18.8–23.5 × 5.3–7.0	2–4	10–25.3	2.5–8
*N. aotearoa*	CBS 367.54	21–28 × 6.5–8.5	2–3	5–12	1.5–4
*N. elaeidis*	MFLUCC 15-0735	10–20 × 3–7	2–3	10–20	(0) 2–6
*N. petila*	MFLUCC 17-1738	21–26.5 × 6–7	2–3	22–29	3–8
*N. piceana*	CBS 394.48	19.5–25 × 7.5–9	3	21–31	6–23
*N. samarangensis*	MFLUCC 12-0233	18–21 × 6.5–7.5	3	12–18	3.5–5.2
***N. rosicola***	NM47-0706	16.9–24.6 × 5.5–7.2	2–4	10–25	1.7–7
*N. rosicola*	CFCC 51992	20.2–25.5 × 5.5–8	2–4	17–22.8	2–9.5
***N. vaccinii***	JR31-0709	14.5–20.6 × 5.5–7.4	2–3	10–22.5	1.3–5.1
*N. vaccinii*	CAA1059	20.9 × 6.4	2–3	8.9–25.3	1.7–6.6
*N. hispanica*	CBS 147686	24.4–25.3 × 7.2–7.8	3–4	19.5–22.6	5.1–15.5
***N. wuzhishanensis***	YX116-0708	19.5–26.5 × 4.5–6.3	1–3	9–20.8	(0) 0.8–3.8
*N. mianyangensis*	UESTCC 22.0006	19–23 × 5.5–7	3	5.5–11	3–4
***N. yongxunensis***	YX101-0708	18.2–25.5 × 5.8–7.5	2–4	10.5–24.7	1.7–7
*N. dendrobii*	MFLUCC 14-0106	20.5–23 × 6.5–7.5	2–3	5–6.5	NA
*N. paeonia-suffruticosa*	CGMCC3.23554	20–23 × 9–11	3–4	22.5–34	3.5–7.5
***P. hydei***	BA11-0708	20.3–27.8 × 4.5–6.6	1–3	3.4–17.2	1–9.5
*P. hydei*	MFLUCC 20-0135	18–35 × 3–6	1–3	3–12	2–8
***Ps. avicenniae***	LF48-0709	20.8–30.7 × 5.8–7.9	1–3	17.2–33.3	2–7.8
*Ps. avicenniae*	MFLUCC 17-0434	22.5–26.5 × 5.5–6	1–3	15.5–28.5	3–4
***Ps. myanmarina***	JR34-0709	25.4–34.8 × 5.8–7.4	2–3	18.1–36.9	2.7–7
*Ps. myanmarina*	NBRC 11226	31–38.5 × 6.5–9	2–3	22.5–38.5	NA

The strains in this study are indicated in bold font. NA: not available.

**Figure 6:**
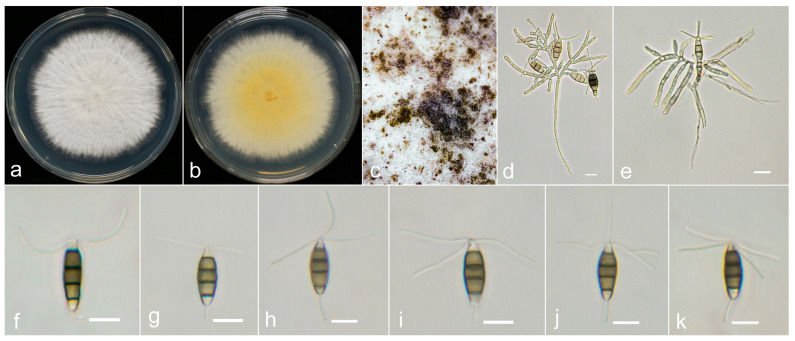
*Neopestalotiopsis oblatespora* (YJ11-0708, holotype). (**a**,**b**) Colony on PDA (above and reverse), (**c**) conidia pile on PDA, (**d**,**e**) conidiophores and (**f**–**k**) conidia. Scale bars = 10 μm.

***Neopestalotiopsis oblatespora*** X.F. Cui and Z.G. Hao, sp. nov. (Figure 6).

MycoBank: MB854051.

**Etymology:** The name refers to the spore morphology.

**Holotype:** YJ11-0708.

**Description:**

Conidiomata were not observed on PDA. The conidiophores are often monopodial, branched and colorless. The conidia are oblate, straight, scarcely curved, 18–23.2 × 5.5–6.7 μm ($\bar{x}$ = 20.2 × 6.2 μm) and have four septa. The basal cell is conical to subcylindrical, pale brown or hyaline, thin and smooth walled and is 2.5–4.5 μm long ($\bar{x}$ = 3.2 μm). The three median cells are 12–15 μm ($\bar{x}$ = 13.6 μm), nearly concolorous or versicolored and brown to dark brown, with the septa and periclinal walls darker than the rest of the cell. The second cell from the base is brown to dark brown and 3.7–6 μm long ($\bar{x}$ = 4.7 μm). The third cell is dark brown and 3–5 μm long ($\bar{x}$ = 4.2 μm). The fourth is dark brown and 3.5–5.3 μm long ($\bar{x}$ = 4.4 μm). The apical cell is 2.5–4 μm long ($\bar{x}$ = 3.2 μm), conical, hyaline, thin and smooth walled. There are 2–4 tubular appendages on the apical cell (often 3) arising from the apex of the apical cell, which are unbranched and 10–26.5 μm long ($\bar{x}$ = 18 μm). The single basal appendage is unbranched, tubular, centric or lateral and 2–9 μm long ($\bar{x}$ = 5.6 μm). A sexual morph was not observed.

**Culture characteristics:** The colonies reached 70 mm in diameter after 4 days on PDA at room temperature, and had serrated-edge, off-white, sparse aerial hyphae on the surface appearing to radiate, turning grey after sporulation.

**Material examined:** The sample originated in China, Hainan Province, Wuzhishan city, Shuiman Township, Yongxun village, from spots on the stem base of *A. oxyphylla*, which was collected on 8 July 2022 by X.F. Cui and Z.G. Hao (YJ11-0708);

**Notes:**

Based on multigene analyses, *Neopestalotiopsis oblatespora* is closely related to *Neopestalotiopsis guajavicola* (FMBCC 11.4), with only a 2 bp difference between them (1/476 in *ITS*; 1/378 in *tef-1α*). However, *N. oblatespora* is distinct from *N. guajavicola*, with a sporulation structure (branched conidiophores of *N. oblatespora* vs. conidiomata of *N. guajavicola*), smaller spores (*N. oblatespora*: 18–23.2 × 5.5–6.7 μm, $\bar{x}$ = 20.2 × 6.2 μm vs. *N. guajavicola* 21.7–24.9 × 6–7 μm, $\bar{x}$ = 23.3 × 6.5 μm) and shorter apical appendages (*N. oblatespora*: 10–26.5 μm, $\bar{x}$ = 18 μm vs. *N. guajavicola*: 19.1–24.5 μm, $\bar{x}$ = 21.8 μm); additionally, *N. oblatespora* has 2–4 apical appendages, while *N. guajavicola* has 2–3 appendages. Moreover, *N. oblatespora* has no significant recombination with its closely taxa according to the PHI test. Therefore, *N. oblatespora* is classified as a new species in the present study.

**Figure 7:**
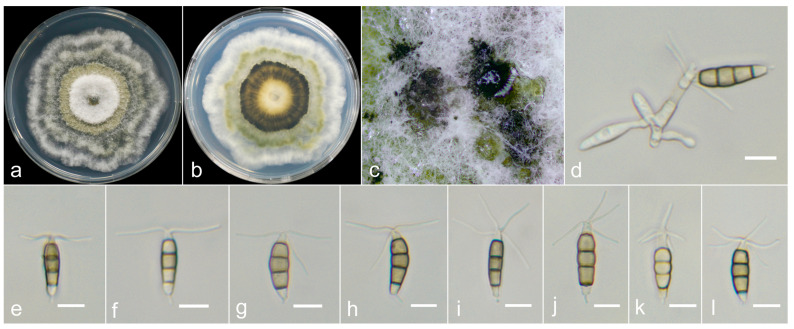
*Neopestalotiopsis olivaceous* (LF25-0709, holotype). (**a**,**b**) Colony on PDA (above and reverse), (**c**) conidia pile on PDA, (**d**) conidiophores and (**e**–**l**) conidia. Scale bars = 10 μm.

***Neopestalotiopsis olivaceous*** X.F. Cui and Z.G. Hao, sp. nov. (Figure 7).

MycoBank: MB854052.

**Etymology:** The name refers to the color of the colony.

**Holotype:** LF25-0709.

**Description:**

Conidiomata were not observed on PDA. The conidia sometimes aggregate, becoming globose, dark green piles. The conidiophore are branched, with spore scars. The conidia are fusiform, straight to obviously irregularly curved, 21.5–33.8 × 5.5–7.7 μm ($\bar{x}$ = 26.5 × 6.3 μm) and have four septa. The basal cell is conical, hyaline or pale olive, smooth, thin walled and 2.7–6.2 μm long ($\bar{x}$ = 4.5 μm). The three median cells are 14 to 21.7 μm long ($\bar{x}$ = 17 μm), pale olivaceous to olivaceous, concolorous and have a rugose wall, with septa darker than the rest of the cell. The second cell from the base is pale olivaceous to olivaceous and 3.3 to 8.5 μm long ($\bar{x}$ = 5.9 μm). The third cell is pale olivaceous to olivaceous and 4 to 6.5 μm long ($\bar{x}$ = 5.1 μm). The fourth cell is pale olivaceous to olivaceous and 4 to 6.5 μm long ($\bar{x}$ = 5.4 μm). The apical cell is 3.5 to 5.5 μm long ($\bar{x}$ = 4.5 μm), hyaline, and conic to acute, with 2 to 5 (often 3–4) tubular appendages on the apical cell, which are inserted at different loci in a crest at the apex of the apical cell, unbranched and 9.5 to 22.5 μm ($\bar{x}$ = 14 μm) long. A single basal appendage, which is occasionally absent, is unbranched, tubular, centric or lateral and 1.2 to 4.8 μm ($\bar{x}$ = 2.4 μm) long. A sexual morph was not observed.

**Culture characteristics:** The colonies reached 70 mm in diameter on PDA after 7 days of growth at room temperature. The colonies appeared circular, white above and medium dense, with aerial hyphae on the flat surface; its reverse was olivaceous, gradually deepening over time.

**Material examined:** The sample originated in China, Hainan Province, Qiongzhong city, Changzheng Township, Luofan village, from leaf spots of *A. oxyphylla*, which was collected on 9 July 2022 by X.F. Cui and Z.G. Hao (LF25-0709, holotype); the ex-type originated in Hainan Province, Baoting city, Shiling Township, Shuixian village, from leaf spots of *A. oxyphylla*, which was collected on 6 July 2022 by X.F. Cui and Z.G. Hao (SX33-0706); the sample originated in Hainan Province, Wuzhishan city, Shuiman Township, Yongxun village, from leaf spots of *A. oxyphylla*, which was collected on 8 July 2022 by X.F. Cui and Z.G. Hao (YX45-0708).

**Notes:** Three strains of *Neopestalotiopsis olivaceous* were isolated from three cities in Hainan, LF25-0709, SX33-0706 and YX45-0708, with well-supported clusters (ML = 99%, BI = 1). *N. olivaceous* clusters a sister group with *N. amomi* (HKAS 124563) and *N. zingiberis* (GUCC 21001). Molecularly, *N. olivaceous* can be differentiated from *N. amomi* (HKAS 124563) and *N. zingiberis* (GUCC 21001), according to *ITS*~*tef-1α*~*tub2* (1/471 of *ITS* and 5/328 of *TEF* with *N. amomi*; 3/447 of *ITS*, 14/719 of *TUB* and 13/358 of *TEF* with *N. zingiberis*). Morphologically, *N. olivaceous* is distinguished with longer conidia (21.5–33.8 μm of *N. olivaceous* vs. 18–30 μm of *N. amomi* and 21–31 μm of *N. zingiberis*), different numbers of apical appendages (2–5 tubular appendages for *N. olivaceous* vs. 2–3 for *N. amomi* and 1–3 for *N. zingiberis*) and longer apical appendages (*N. olivaceous* with 9.5–22.5 μm vs. *N. amomi* with 7–17 μm and *N. zingiberis* with 12–15 μm). The results of the PHI test showed no significant recombination among *N. olivaceous* and its closely related taxa. Thus, *N. olivaceous* is classified as a new species in the present study.

**Figure 8:**
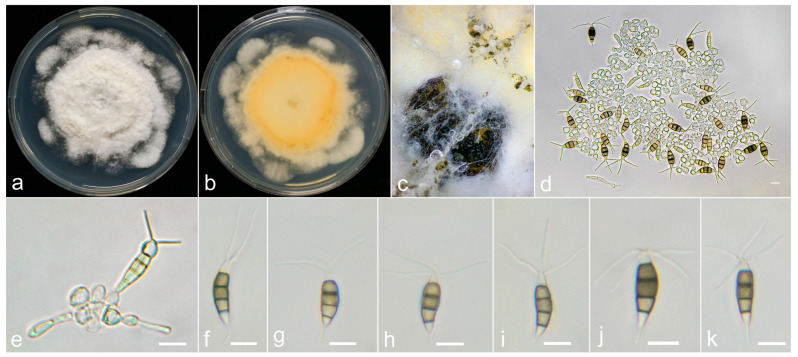
*Neopestalotiopsis oxyphylla* (LF55-0709, holotype). (**a**,**b**) Colony on PDA (above and reverse), (**c**) conidiomata on PDA, (**d**,**e**) conidiogenous cells, and (**f**–**k**) conidia. Scale bars = 10 μm.

***Neopestalotiopsis oxyphylla*** X.F. Cui and Z.G. Hao, sp. nov. (Figure 8).

MycoBank: MB854053.

**Etymology:** It is named in reference to the host species, *Alpinia oxyphylla*.

**Holotype:** LF55-0709.

**Description:**

The conidiomata are solitary or aggregated, globose and dark, often immersed in PDA. The conidiophores are distinct, often degenerated to conidiogenous cells. Conidiogenous cells are spherical and hyaline. The conidia are fusiform, straight to slightly curved, 18.8–23.5 × 5.3–7.0 μm ($\bar{x}$ = 21 × 6.2 μm) and have four septa. The basal cell is conical to subcylindrical, hyaline, thin and smooth walled and is 2.3–5 μm long ($\bar{x}$ = 3.9 μm). The three median cells are 11.3–15 μm ($\bar{x}$ = 13 μm), versicolored and brown to dark brown, with septa and periclinal walls that are darker than the rest of the cell and a wall with verrucae. The second cell from the base is pale brown, paler than the other two cells and 3.3–5.2 μm long ($\bar{x}$ = 4.1 μm). The third cell is dark brown, darker than the other two and 3.5–5.0 μm long ($\bar{x}$ = 4.1 μm); the fourth is pale brown to brown and 3.7–5.4 μm long ($\bar{x}$ = 4.4 μm). The apical cell is 2.8–5 μm long ($\bar{x}$ = 3.8 μm), conic to acute, hyaline, thin and smooth walled, with 2–4 tubular appendages on the apical cell (often 2–3) arising from the apex of the apical cell, which are occasionally branched, flexuous and 10–25.3 μm long ($\bar{x}$ = 18.6 μm). The single basal appendage is unbranched, tubular, centric and 2.5–8 μm long ($\bar{x}$ = 5 μm). A sexual morph was not observed.

**Culture characteristics:** The colonies reached 70 mm in diameter after 9 days on PDA at room temperature, with circular-edge, off-white, dense, central aerial hyphae on the raised surface, with a filiform margin, black fruiting bodies and a reverse similar in color.

**Material examined:** The sample originated in China, Hainan Province, Qiongzhong city, Changzheng Township, Luofan village, from leaf spots of *A. oxyphylla*, which was collected on 9 July 2022 by X.F. Cui and Z.G. Hao (LF55-0709, holotype); the ex-type was from Hainan Province, Wuzhishan city, Maoyang Township, Maohui village, from stem base spots of *A. oxyphylla*, which was collected on 8 July 2022 by X.F. Cui and Z.G. Hao (MJ31-0708); the ex-type originated in Hainan Province, Baoting city, Nanmao Shengli Farm, from leaf spots of *A. oxyphylla*, which was collected on 6 July 2022 by X.F. Cui and Z.G. Hao (NM44-0706).

**Notes:**

Based on multigene analyses, *Neopestalotiopsis oxyphylla* is closely related to *N. brachiata* (MFLUCC 17-1555), *N. elaeidis* (MFLUCC 15-0735), *N. petila* (MFLUCC 17-1738), *N. aotearoa* (CBS 367.54) and *N. piceana* (CBS 394.48), with only 0–2 bp difference among them. However, *N. oxyphylla* is distinct from *N. elaeidis*, with larger spores (*N. oxyphylla*: 18.8–23.5 × 5.3–7.0 μm, $\bar{x}$ = 21 × 6.2 μm vs. *N. elaeidis* 10–20 × 3–7 μm, $\bar{x}$ = 16 × 5.5 μm) and thinner spores (*N. oxyphylla*: 5.3–7.0 μm vs. *N. aotearoa*: 6.5–8.5 μm and *N. piceana* 7.5–9 μm); *N. oxyphylla* has different numbers of apical appendages (*N. oxyphylla:* 2–4; *N. brachiata:* 1–3; *N. aotearoa, N. elaeidis* and *N. petila:* 2–3; *N. piceana:* 3) and shorter apical appendages (*N. oxyphylla:* 10–25.3 μm vs. *N. brachiata:* 9.5–33; *N. petila*: 22–29 μm, *N. piceana*: 21–31 μm), but they are longer than those of *N. aotearoa* (5–12 μm). In addition, *N. oxyphylla* has shorter basal appendages (*N. oxyphylla*: 2.5–8 μm vs. *N. piceana*: 6–23 μm). Therefore, *N. oxyphylla* is classified as a new species in the present study.

**Figure 9:**
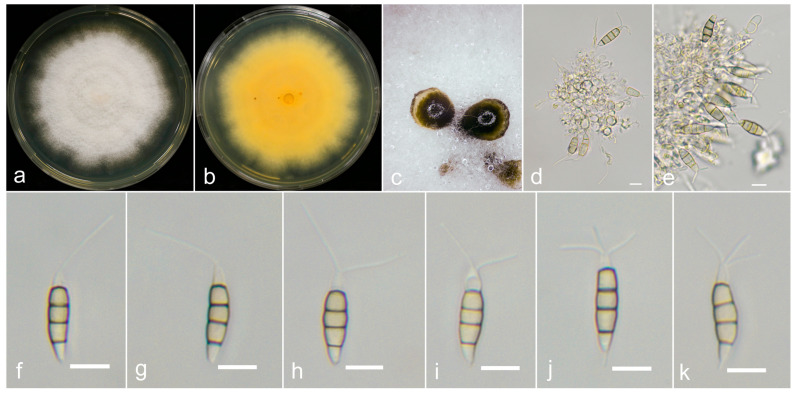
*Neopestalotiopsis wuzhishanensis* (YX116-0708, holotype). (**a**,**b**) Colony on PDA (above and reverse), (**c**) conidiomata on PDA, (**d**,**e**) conidiogenous cells and (**f**–**k**) conidia. Scale bars = 10 μm.

***Neopestalotiopsis wuzhishanensis*** X.F. Cui and Z.G. Hao, sp. nov. (Figure 9).

MycoBank: MB854054.

**Etymology:** It is named in reference to the first collection city of Wuzhishan in Hainan province.

**Holotype:** YX116-0708.

**Description:**

The conidiomata on the PDA are solitary, globose and dark. The conidiophores often degenerated to conidiogenous cells. Conidiogenous cells are unclear. The conidia arefusiform, straight, scarcely curved, 19.5–26.5 × 4.5–6.3 μm ($\bar{x}$ = 22.4 × 5.2 μm) and have four septa. The basal cell is conical to subcylindrical, hyaline, thin and smooth walled and is 2.8–5.5 μm long ($\bar{x}$ = 4.2 μm). The three median cells are 12.8–16 μm ($\bar{x}$ = 14.4 μm), nearly concolorous, pale brown and hyaline, with septa and periclinal walls darker than the rest of the cell. The second cell from the base is pale brown and 4–6.2 μm long ($\bar{x}$ = 5.1 μm). The third cell is pale brown and 3.5–5.2 μm long ($\bar{x}$ = 4.4 μm); the fourth is pale brown and 3.8–6.3 μm long ($\bar{x}$ = 4.7 μm). The apical cell is 2.7–5.5 μm long ($\bar{x}$ = 3.6 μm), conic to acute, hyaline, thin and smooth-walled, with 1–3 tubular appendages on the apical cell (often 1–2) arising from the apex of the apical cell, which are unbranched, straight to flexuous and 9–20.8 μm long ($\bar{x}$ = 15.4 μm). There is a single or no basal appendage, which is unbranched, tubular, centric and 0.8–3.8 μm long ($\bar{x}$ = 1.9 μm). A sexual morph is not observed.

**Culture characteristics:** The colonies reached 70 mm in diameter after 12 days on PDA at room temperature, with circular-edge, white, medium-dense, aerial hyphae on the flat surface, with a filiform margin, black and fruiting bodies. And its reverse was lemon yellow.

**Material examined:** The sample originated in China, Hainan Province, Wuzhishan city, Shuiman Township, Yongxun village, from leaf spots of *A. oxyphylla*, which was collected on 8 July 2022 by X.F. Cui and Z.G. Hao (YX116-0708).

**Notes:**

*Neopestalotiopsis wuzhishanensis* clusters a sister group to *Neopestalotiopsis cubana* (CBS 600.96), while *N. wuzhishanensis* is different from *N. cubana* depending on *ITS*, *tef-1α* and *tub2* sequences (3/481 in *ITS*, 2/434 in *tef-1α* and 3/715 in *tub2*). Additionally, there are remarkabe discrepancies in the morphological characteristics: *N. wuzhishanensis* is thinner (*N. wuzhishanensis*: 4.5–6.3 μm, $\bar{x}$ = 5.2 μm vs. *N. cubana* 8–9.5 μm, $\bar{x}$ = 8.8 μm) and shorter in its apical appendages (*N. wuzhishanensis*: 9–20.8 μm, $\bar{x}$ = 15.4 μm vs. *N. cubana*: 21–27 μm, $\bar{x}$ = 24 μm) and base appendage (*N. wuzhishanensis*: 0.8–3.8 μm, $\bar{x}$ = 1.9 μm vs. *N. cubana*: 4–7 μm); additionally, the three median cells of *N. wuzhishanensis* are paler than *N. cubana*; furthermore, *N. cubana* has 1–3 apical appendages, while *N. cubana* carries 2–4 appendages. The results of the PHI test showed that *N. wuzhishanensis* has no significant recombination with its closely related taxa. Therefore, *N. wuzhishanensis* is classified as a new species in the present study.

**Figure 10:**
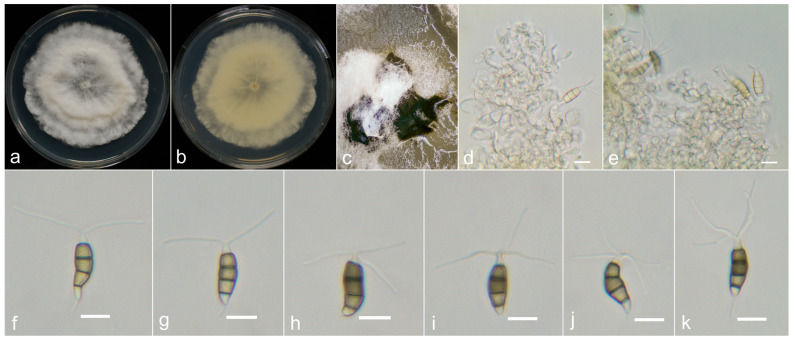
*Neopestalotiopsis yongxunensis* (YX101-0708, holotype). (**a**,**b**) Colony on PDA (above and reverse), (**c**) conidiomata on PDA, (**d**,**e**) conidiogenous cells and (**f**–**k**) conidia. Scale bars = 10 μm.

***Neopestalotiopsis yongxunensis*** X.F. Cui and Z.G. Hao, sp. nov. (Figure 10).

MycoBank: MB854055.

**Etymology:** The name refers to the first collection village of Yongxun in Hainan Province.

**Holotype:** YX101-0708.

**Description:**

The conidiomata on the PDA are solitary or aggregated, globose, dark and embedded or semi-immersed. The conidiophores often degenerated to conidiogenous cells. Conidiogenous cells are unclear. The conidia are fusiform, straight to curved, 18.2–25.5 × 5.8–7.5 μm ($\bar{x}$ = 21.6 × 6.6 μm) and have four septa. The basal cell is conical, hyaline, thin and smooth walled and is 3.0–5.2 μm long ($\bar{x}$ = 4.1 μm). The three median cells are 12–15.2 μm ($\bar{x}$ = 13.7 μm), versicolored, pale brown to brown and have septa and periclinal walls that are darker than the rest of the cell; the second cell from the base is pale brown, paler than the other two cells and is 3.5–5.3 μm long ($\bar{x}$ = 4.3 μm). The third cell is brown, darker than the other two and is 3.8–5.3 μm long ($\bar{x}$ = 4.6 μm). The fourth cell is brown and 4.0–5.2 μm long ($\bar{x}$ = 4.6 μm). The apical cell is 2.5–5.0 μm long ($\bar{x}$ = 3.7 μm), conic to subcylindrical, hyaline, thin and smooth walled, with 2–4 tubular appendages on the apical cell arising from the apex of the apical cell, which are filiform, unbranched, straight to flexuous and 10.5–24.7 μm long ($\bar{x}$ = 18.2 μm). The single basal appendage is unbranched, tubular, centric and 1.7–7 μm long ($\bar{x}$ = 4.2 μm). A sexual morph is not observed.

**Culture characteristics:** The colonies reached 70 mm in diameter after 4 days on PDA at room temperature, with a circular-edge, white, dense aerial mycelium on the surface; the reverse was similar in color. The fruiting bodies were black, mostly under the hyphae and were visible on the back.

**Material examined:** The sample originated in China, Hainan Province, Wuzhishan city, Shuiman Township, Yongxun village, from leaf spots of *A. oxyphylla*, which was collected on 8 July 2022 by X.F. Cui and Z.G. Hao (YX101-0708);

**Notes:**

*Neopestalotiopsis yongxunensis* is related to *N. dendrobii* (MFLUCC 14-0106) and *N. paeonia-suffruticosa* (HKAS 123212), as shown in the phylogenetic analysis, while *N. yongxunensis* can be differentiated from *N. dendrobii* and *N. paeonia-suffruticosa* depending on *ITS*, *tef1-α* and *tub2* sequences, showing a 7 bp difference (2/284 in *tef1-α* and 5/416 in *tub2*) with *N. dendrobii* and 10 bp difference (9/440 in *tef1-α* and 1/715 in *tub2*) with *N. paeonia-suffruticosa*. In addition, there are remarkable discrepancies in the morphological characteristics: *N. yongxunensis* is thinner in its conidia (*N. yongxunensis*: 5.8–7.5 μm, $\bar{x}$ = 6.6 μm vs. *N. paeonia-suffruticosa*: 9–11 μm, $\bar{x}$ = 9.5 μm), has different numbers of apical appendages (*N. yongxunensis* 2–4 vs. *N. paeonia-suffruticosa* 3–4) and shorter apical appendages (*N. yongxunensis*: 10.5–24.7 μm vs. *N. paeonia-suffruticosa* 22.5–34 μm), while *N. yongxunensis* differs from *N. dendrobii* in having longer apical appendages (*N. yongxunensis*: 10.5–24.7 μm vs. *N. dendrobii* 5–6.5 μm) with different numbers (*N. yongxunensis* 2–4 vs. *N. dendrobii* 2–3). Furthermore, the PHI test indicated that there is no significant recombination between *N. yongxunensis* and its closely related species. Therefore, *N. yongxunensis* is classified as a new species in the present study.

### 3.4. Pathogenicity Assay

**Figure 11:**
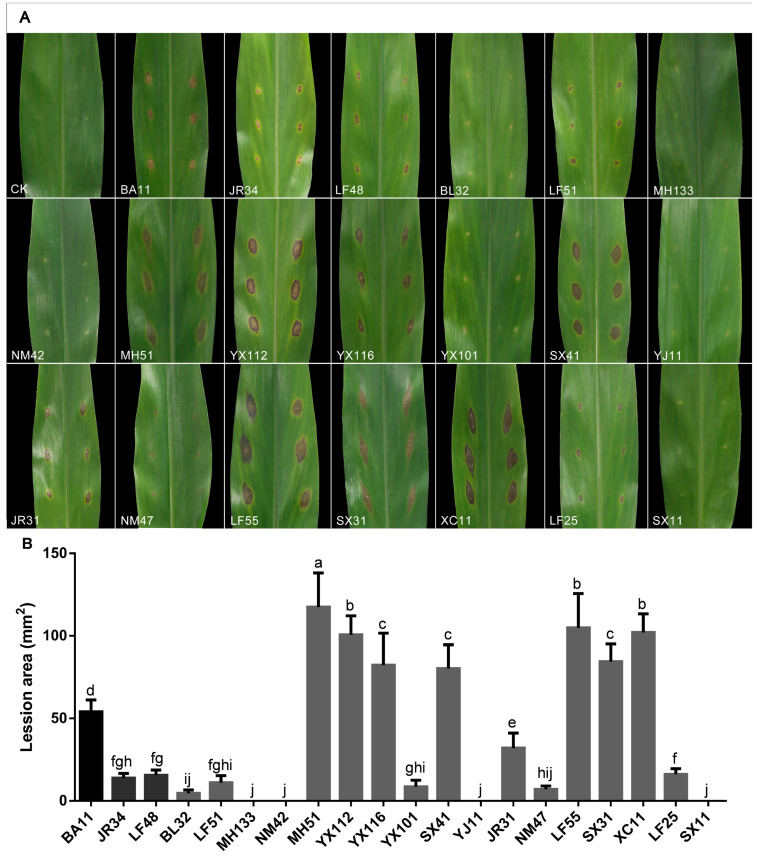
Pathogenicity test results of 20 pestalotioid species on *Alpinia oxyphylla* leaves. (**A**) Symptoms on leaves after 5 days. Icons in figures, in sequence, are CK, *P. hydei* BA11-0708, *Ps. myanmarina* JR34-0709, *Ps. avicenniae* LF48-0709, *N. coffeae-arabicae* BL32-0708, *N. coffeae-arabicae* LF51-0709, *Neopestalotiopsis* sp.4 MH133-0708, *N. coffeae-arabicae* NM42-0706, *N. cubana* MH51-0708, *N. cubana* YX112-0708, *N. wuzhishanensis* YX116-0708, *N. yongxunensis* YX101-0708, *N. baotingensis* SX41-0706, *N. oblatespora* YJ11-0708, *N. vaccinii* JR31-0709, *N. rosicola* NM47-0708, *N. oxyphylla* LF55-0709, *N. brachiata* SX31-0706, *Neopestalotiopsis* sp.5 XC11-0709, *N. olivaceous* LF25-0709 and *Neopestalotiopsis* sp.3 SX11-0706. (**B**) Pathogenicity of the isolates was evaluated by measuring the area of the necrotic lesions after 5 days. Error bars indicate the standard deviation of the mean. Significant differences (*p* < 0.05) are indicated with different letters according to Duncan’s multiple range test. The abscissa designation corresponds sequentially to (**A**), excluding “CK”.

Sixteen of the twenty tested pestalotioid isolates were able to cause typical brown lesions after inoculation, while the other four isolates did not, including *Neopestalotiopsis* sp.4 MH133-0708, *N. coffeae-arabicae* NM42-0706, *N. oblatespora* YJ11-0708 and *Neopestalotiopsis* sp.3 SX11-0706. The lesion areas measured 5 days after inoculation were 54.02, 13.86, 15.57, 4.65, 11.08, 117.40, 100.63, 82.31, 8.55, 80.25, 32.03, 7.02, 104.86, 84.48, 102.04 and 16.17 mm^2^ for isolates of *P. hydei* BA11-0708, *Ps. myanmarina* JR34-0709, *Ps. avicenniae* LF48-0709, *N. coffeae-arabicae* BL32-0708, *N. coffeae-arabicae* LF51-0709, *N. cubana* MH51-0708, *N. cubana* YX112-0708, *N. wuzhishanensis* YX116-0708, *N. yongxunensis* YX101-0708, *N. baotingensis* SX41-0706, *N. vaccinii* JR31-0709, *N. rosicola* NM47-0706, *N. oxyphylla* LF55-0709, *N. brachiata* SX31-0706, *Neopestalotiopsis* sp.5 XC11-0709 and *N. olivaceous* LF25-0709, respectively (Figure 11). The morphology of the purified fungi re-isolated from the lesion after inoculation was identical with those of the isolates used for inoculation, which were also confirmed using PCR and gene sequences. The results of the pathogenicity and phylogenetic analysis showed that the strains close to *N. cubana* and *N. brachiata* had a stronger pathogenicity (Figure 1 and Figure 11B).

## 4. Discussion

In this study, 36 *pestalotioid* strains were isolated. According to the multi-locus phylogeny (*ITS*, *tef-1α* and *tub2*) and morphological characteristics analyses, one *Pestalotiopsis* sp, two *Pseudopestalotiopsis* spp., and fourteen *Neopestalotiopsis* spp. were identified. Six new species (*N. baotingensis, N. oblatespora, N. olivaceous*, *N. oxyphylla*, *N. wuzhishanensis* and *N. yongxunensis*) were described. Among the 36 strains, the isolation frequency of *N. coffeae-arabicae* and *N. cubana* was 16.67% for both, higher than the others; additionally, *N. coffeae-arabicae*, *N. olivaceous* and *N. oxyphylla* were isolated from five, three and three cities separately, with a wider distribution in Hainan than others. This is the first systematic report of *Neopestalotiopsis*, *Pestalotiopsis* and *Pseudopestalotiopsis* fungi relating to *A. oxyphylla* in its main planted area.

The development of molecular biology has greatly facilitated the identification of microorganisms, and the phylogeny analyses of combined *ITS*, *tef-1α* and *tub2* can better distinguish *Neopestalotiopsis*, *Pestalotiopsis* and *Pseudopestalotiopsis*. For example, in this study, *N. olivaceous* and *N. wuzhishanensis* do not conform to the morphological characteristics of the versicolorous median cells depicted in *Neopestalotiopsis*. This phenomenon was also mentioned by Sun et al. [18], so the phylogeny analyses can overcome the discrimination of the three genera only by the intermediate cell color. While the three gene sequences of *N. oxyphylla*, *N. aotearoa* and *N. brachiata* are closely similar, only with 0–2 bp difference in the combined sequence, and between *N. oblatespora* and *N. guajavicola*, with 2 bp difference, they have obvious discrepancies in their morphological characteristics. A similar phenomenon was observed between *N. alpapicalis* MFLUCC 17-2544^T^ and *N. rhizophorae* MFLUCC 17-1551^T^ [41]. Therefore, more gene fragments need to be introduced in order to further differentiate closely related species of pestalotioid fungi.

RLB is an important disease in the cultivation process of *A. oxyphylla,* according to previous reports. Its pathogen was reported as *Pestalotia palmarum* in 1986 [11], now classified as *Pestalotiopsis palmarum,* while the RBS disease, with similar symptoms to RLB disease, was caused by *Pestalosphaeria alpinia,* the sexual morph of pestalotioid, as reported in 1994 [15]. Perhaps due to the differences in the classification method and limitations in the sample size, *P. palmarum* and *P. alpinia* were not isolated in this study, which explained the potential diversity of pestalotioid fungi in this host that need to be further explored. In addition, the symptoms of RLB and RBS disease are similar, with both caused by pestalotioid fungi with different morphs, so it is recommended to merge the two diseases into one for future research and disease management.

The pathogenicity tests of 20 pestalotioid strains showed that most species can cause obvious symptoms on the leaves, indicating the diversity of the pathogen of RLB disease, and the *Neopestalotiopsis* species (lesion area over 75 mm^2^ for 7 species) tended to infect *A. oxyphylla* and caused more serious disease than *Pestalotiopsis* (lesion area of about 50 mm^2^) and *Pseudopestalotiopsis* (less than 50 mm^2^ for both). The reports of the disease caused by *Neopestalotiopsis* fungi have been more frequent in recent years [18]. In addition, all pathogenicity tests were carried out with a single cultivar of *A. oxyphylla* and constant environmental conditions. As we all know, both differences in varieties and changes in the environmental conditions can affect the occurrence of diseases. Therefore, more studies need to be performed on different varieties under different environmental conditions.

What is worth noting is that most of the pestalotioid species have a broad range of hosts, and one species of pestalotioid fungi can infect several economic plants, while a plant can be harmed by several pestalotioid fungi. For example, *N. cubana* can infect rubber trees [96], *Camellia oleifera* [19] and *Ixora chinensis* [97], and a new leaf fall disease of rubber trees was caused by *N. aotearoa*, *N. cubana* and *N. formicarum* [96]. *A. oxyphylla* is a semi-shade plant mainly planted in rubber tree forests. In this study, six strains of *N. cubana*, one strain of *Neopestalotiopsis* sp.3 SX11-0706 clustered with *N. formicarum* and five strains (*N. oxyphylla*, *N. brachiata* and *Neopestalotiopsis* sp.5 XC11-0709) closely related to *N. aotearoa* were isolated. Thus, it suggests that some pestalotioid species may infect both the rubber tree and *A. oxyphylla*. The promotion of medicinal plant cultivation under forest trees should be carried out with attention to the occurrence of cross-infection diseases in order to prevent them.

A comprehensive understanding of the species and genetic diversity of pathogens is the foundation for sustainable disease management. Since there is no research about the resistance varieties of *A. oxyphylla* to RLB disease, the strains with different characteristics and pathogenicities that were isolated in this study may provide a material basis for the subsequent screening of resistant varieties, including highly active biological and chemical agents friendly to the environment.

## Figures and Tables

**Table 1:** PCR primers and procedures used in this study.

Locus	Primes Name	Sequence (5′ to 3′)	PCR Procedures	Reference
*ITS*	ITS5	GGAAGTAAAAGTCGTAACAAGG	95 °C for 5 min; 94 °C for 25 s; 52 °C for 25 s; 72 °C for 10 s; repeat 2 to 4 for 35 cycles; 72 °C for 5 min; 4 °C on hold	[35]
	ITS4	TCCTCCGCTTATTGATATGC		
*tef-1α*	EF1-728F	CATCGAGAAGTTCGAGAAGG	95 °C for 5 min; 94 °C for 25 s; 52 °C for 25 s; 72 °C for 10 s (15 s); repeat 2 to 4 for 35 cycles; 72 °C for 5 min; 4 °C on hold	[36,37]
	EF1-526F	GTCGTYGTYATYGGHCAYGT		
	EF2	GGARGTACCAGTSATCATGTT		
*tub2*	T1	AACATGCGTGAGATTGTAAGT	95 °C for 5 min; 94 °C for 25 s; 55 °C for 25 s; 72 °C for 15 s; repeat 2 to 4 for 35 cycles; 72 °C for 5 min; 4 °C on hold	[38,39]
	Bt2b	ACCCTCAGTGTAGTGACCCTTGGC		

**Table 2:** The strain information and gene accession numbers for pestalotioid species used in this study.

Taxonomic Status	Strain No.	Host/Substrate	Origin	GenBank Accession Numbers			References
				ITS	*tub 2*	*tef-lα*	
*Neopestalotiopsis acrostichi*	MFLUCC 17-1754^T^	*Acrostichum aureum*	Thailand	MK764272	MK764338	MK764316	[41]
*N. acrostichi*	MFLUCC 17-1755	*Acrostichum aureum*	Thailand	MK764273	MK764339	MK764317	[41]
*N. alpapicalis*	MFLUCC 17-2544^T^	*Rhizophora mucronata*	Thailand	MK357772	MK463545	MK463547	[42]
*N. alpapicalis*	MFLUCC 17-2545	*Rhizophora mucronata*	Thailand	MK357773	MK463546	MK463548	[42]
*N. amomi*	HKAS 124563^T^	*Amomum villosum*	China	OP498012	OP752133	OP653489	[18]
*N. amomi*	HKAS 124564	*Amomum villosum*	China	OP498013	OP765913	OP753382	[18]
*N. aotearoa*	CBS 367.54^T^	Canvas	New Zealand	KM199369	KM199454	KM199526	[17]
*N. asiatica*	MFLUCC 12-0286^T^	*Prunus dulcis*	China	JX398983	JX399018	JX399049	[17]
*N. australis*	CBS 114159^T^	*Telopea* sp.	Australia	KM199348	KM199432	KM199537	[17]
***N. baotingensis***	**SX41-0706^T^**	***Alpinia oxyphylla***	**China**	**PP621751**	**PP767811**	**PP767847**	**In this study**
***N. baotingensis***	**YJ34-0708**	***Alpinia oxyphylla***	**China**	**PP621765**	**PP767815**	**PP767851**	**In this study**
*N. brachiata*	MFLUCC 17-1555	*Rhizophora apiculata*	Thailand	MK764274	MK764340	MK764318	[41]
***N. brachiata***	**SX31-0706**	***Alpinia oxyphylla***	**China**	**PP621749**	**PP767809**	**PP767845**	**In this study**
*N. brasiliensis*	COAD 2166^T^	*Psidium guajava*	Brazil	MG686469	MG692400	MG692402	[43]
*N. brasiliensis*	CFCC 54341	*Castanea mollissima*	China	MW166229	MW218522	MW199748	[44]
*N. camelliae-oleiferae*	CSUFTCC81^T^	*Camellia oleifera*	China	OK493585	OK562360	OK507955	[19]
*N. camelliae-oleiferae*	CSUFTCC82	*Camellia oleifera*	China	OK493586	OK562361	OK507956	[19]
*N. cavernicola*	KUMCC 20-0269^T^	Cave	China	MW545802	MW557596	MW550735	[45]
*N. chiangmaiensis*	MFLUCC 18–0113	*Pandanus* sp.	Thailand	NA	MH412725	MH388404	[46]
*N. chrysea*	MFLUCC 12-0261^T^	Dead leaves	China	JX398985	JX399020	JX399051	[16]
*N. chrysea*	MFLUCC 12-0262	Dead leaves	China	JX398986	JX399021	JX399052	[16]
*N. clavispora*	MFLUCC 12-0281^T^	*Magnolia* sp.	China	JX398979	JX399014	JX399045	[16]
*N. clavispora*	MFLUCC 12-0280	*Magnolia* sp.	China	JX398978	JX399013	JX399044	[16]
*N. cocoes*	MFLUCC 15-0152^T^	*Cocos nucifera*	Thailand	KX789687	NA	KX789689	[41]
*N. coffeae-arabicae*	HGUP4015	*Coffea arabica*	China	KF412647	KF412641	KF412644	[47]
*N. coffeae-arabicae*	HGUP4019^T^	*Coffea arabica*	China	KF412649	KF412643	KF412646	[47]
***N. coffeae-arabicae***	**BL32-0708**	***Alpinia oxyphylla***	**China**	**PP621754**	**PP767790**	**PP767826**	**In this study**
***N. coffeae-arabicae***	**JR23-0709**	***Alpinia oxyphylla***	**China**	**PP621735**	**PP767791**	**PP767827**	**In this study**
***N. coffeae-arabicae***	**LF26-0709**	***Alpinia oxyphylla***	**China**	**PP621742**	**PP767797**	**PP767833**	**In this study**
***N. coffeae-arabicae***	**LF33-0709**	***Alpinia oxyphylla***	**China**	**PP621743**	**PP767798**	**PP767834**	**In this study**
***N. coffeae-arabicae***	**LF51-0709**	***Alpinia oxyphylla***	**China**	**PP621745**	**PP767799**	**PP767835**	**In this study**
***N. coffeae-arabicae***	**NM42-0706**	***Alpinia oxyphylla***	**China**	**PP621767**	**PP767804**	**PP767840**	**In this study**
*N. concentrica*	CFCC 55162^T^	*Rosa chinensis*	China	OK560707	OM117698	OM622433	[48]
*N. cubana*	CBS 600.96^T^	Leaf litter	Cuba	KM199347	KM199438	KM199521	[17]
***N. cubana***	**MH51-0708**	***Alpinia oxyphylla***	**China**	**PP621752**	**PP767802**	**PP767838**	**In this study**
***N. cubana***	**NM48-0706**	***Alpinia oxyphylla***	**China**	**PP621770**	**PP767807**	**PP767843**	**In this study**
***N. cubana***	**YJ33-0708**	***Alpinia oxyphylla***	**China**	**PP621764**	**PP767814**	**PP767850**	**In this study**
***N. cubana***	**YX112-0708**	***Alpinia oxyphylla***	**China**	**PP621761**	**PP767817**	**PP767853**	**In this study**
***N. cubana***	**YX41-0708**	***Alpinia oxyphylla***	**China**	**PP621757**	**PP767819**	**PP767855**	**In this study**
***N. cubana***	**YX44-0708**	***Alpinia oxyphylla***	**China**	**PP621758**	**PP767820**	**PP767856**	**In this study**
*N. dendrobii*	MFLUCC 14-0106^T^	*Dendrobium cariniferum*	Thailand	MK993571	MK975835	MK975829	[49]
*N. dendrobii*	MFLUCC 14-0099	*Dendrobium cariniferum*	Thailand	MK993570	MK975834	MK975828	[49]
*N. drenthii*	BRIP 72263a	*Macadamia integrifolia*	Australia	MZ303786	MZ312679	MZ344171	[22]
*N. drenthii*	BRIP 72264a^T^	*Macadamia integrifolia*	Australia	MZ303787	MZ312680	MZ344172	[22]
*N. egyptiaca*	CBS 140162^T^	*Mangifera indica*	Egypt	KP943747	KP943746	KP943748	[50]
*N. elaeagni*	HGUP10002^T^	*Elaeagnus pungens*	China	MW930716	MZ683391	MZ203452	[30]
*N. elaeidis*	MFLUCC 15-0735	*Elaeis guineensis*	Thailand	ON650689	NA	ON734012	[51]
*N. ellipsospora*	MFLUCC 12-0283^T^	Dead plant	China	JX398980	JX399016	JX399047	[16]
*N. eucalyptorum*	CBS 147684^T^	*Eucalyptus globulus*	Portugal	MW794108	MW802841	MW805397	[20]
*N. eucalypticola*	CBS 264.37^T^	*Eucalyptus globulus*	NA	KM199376	KM199431	KM199551	[17]
*N. fragariae*	ZHKUCC 22-0115	*Fragaria* x *ananassa*	China	ON651146	ON685199	ON685197	[32]
*N. foedans*	CGMCC 3.9123^T^	Mangrove plant	China	JX398987	JX399022	JX399053	[16]
*N. foedans*	CGMCC 3.9178	*Neodypsis decaryi*	China	JX398989	JX399024	JX399055	[16]
*N. formicarum*	CBS 362.72^T^	Dead ant	Cuba	KM199358	KM199455	KM199517	[17]
*N. formicarum*	CBS 115.83	Plant debris	Cuba	KM199344	KM199444	KM199519	[17]
*N. guajavae*	FMBCC 11.1^T^	*Psidium guajava*	Pakistan	MF783085	MH460871	MH460868	[52]
*N. guajavicola*	FMBCC 11.4^T^	*Psidium guajava*	Pakistan	MH209245	MH460873	MH460870	[52]
*N. haikouensis*	SAUCC212271^T^	*Ilexchinensis* sp.	China	OK087294	OK104870	OK104877	[53]
*N. hadrolaeliae*	COAD 2637^T^	*Hadrolaelia jongheana*	Brazil	MK454709	MK465120	MK465122	[54]
*N. hispanica*	CBS 147686^T^	*Eucalyptus globulus*	Portugal	MW794107	MW802840	MW805399	[20]
*N. honoluluana*	CBS 114495^T^	*Telopea* sp.	USA	KM199364	KM199457	KM199548	[17]
*N. hydeana*	MFLUCC 20-0132^T^	*Artocarpus heterophyllus*	Thailand	MW266069	MW251119	MW251129	[55]
*N. hyperici*	HKAS 124561	*Hypericum monogynum*	China	OP498010	OP765908	OP713768	[18]
*N. iberica*	CBS 147688^T^	*Eucalyptus globulus*	Portugal	MW794111	MW802844	MW805402	[20]
*N. javaensis*	CBS 257.31^T^	*Cocos nucifera*	Indonesia	KM199357	KM199437	KM199543	[17]
*N. lusitanica*	CBS 147690^T^	*Eucalyptus globulus*	Portugal	MW794110	MW802843	MW805406	[20]
*N. longiappendiculata*	CBS 147692^T^	*Eucalyptus globulus*	Portugal	MW794112	MW802845	MW805404	[20]
*N. macadamiae*	BRIP 63737c^T^	*Macadamia integrifolia*	Australia	KX186604	KX186654	KX186627	[56]
*N. macadamiae*	BRIP 63742a	*Macadamia integrifolia*	Australia	KX186599	KX186657	KX186629	[56]
*N. maddoxii*	BRIP 72266a^T^	*Macadamia integrifolia*	Australia	MZ303782	MZ312675	MZ344167	[22]
*N. magna*	MFLUCC 12-0652^T^	*Pteridium* sp.	France	KF582795	KF582793	KF582791	[57]
*N. mesopotamica*	CBS 336.86^T^	*Pinus brutia*	Iraq	KM199362	KM199441	KM199555	[17]
*N. mesopotamica*	CBS 299.74	*Eucalyptus* sp.	Turkey	KM199361	KM199435	KM199541	[17]
*N. mianyangensis*	HKAS 123211	*Paeonia suffruticosa*	China	OP546681	OP672161	OP723490	[31]
*N. musae*	MFLUCC 15-0776^T^	*Musa* sp.	Thailand	KX789683	KX789686	KX789685	[41]
*N. natalensis*	CBS 138.41^T^	*Acacia mollissima*	South Africa	KM199377	KM199466	KM199552	[17]
*N. nebuloides*	BRIP 66617^T^	*Sporobolus elongatus*	Australia	MK966338	MK977632	MK977633	[58]
***N. oblatespora***	**YJ11-0708^T^**	***Alpinia oxyphylla***	**China**	**PP621763**	**PP767813**	**PP767849**	**In this study**
***N. olivaceous***	**LF25-0709^T^**	***Alpinia oxyphylla***	**China**	**PP621741**	**PP767796**	**PP767832**	**In this study**
***N. olivaceous***	**SX33-0706**	***Alpinia oxyphylla***	**China**	**PP621750**	**PP767810**	**PP767846**	**In this study**
***N. olivaceous***	**YX45-0708**	***Alpinia oxyphylla***	**China**	**PP621759**	**PP767821**	**PP767857**	**In this study**
*N. olumideae*	BRIP 72273a^T^	*Macadamia integrifolia*	Australia	MZ303790	MZ312683	MZ344175	[22]
***N. oxyphylla***	**LF55-0709^T^**	***Alpinia oxyphylla***	**China**	**PP621746**	**PP767800**	**PP767836**	**In this study**
***N. oxyphylla***	**MJ31-0708**	***Alpinia oxyphylla***	**China**	**PP621766**	**PP767803**	**PP767839**	**In this study**
***N. oxyphylla***	**NM44-0706**	***Alpinia oxyphylla***	**China**	**PP621768**	**PP767805**	**PP767841**	**In this study**
*N. paeoniea*	CBS 318.74	*Anacardium occidentale*	Nigeria	MH554031	MH554707	NA	[59]
*N. paeonia-suffruticosa*	HKAS 123212^T^	*Paeonia suffruticosa*	China	OP082292	OP235980	OP204794	[31]
*N. pernambucana*	URM 7148-01^T^	*Vismia guianensis*	Brazil	KJ792466	NA	KU306739	[60]
*N. perukae*	FMBCC11.3^T^	Guava	Pakistan	MH209077	MH460876	MH523647	[52]
*N. petila*	MFLUCC 17-1737	*Rhizophora mucronata*	Thailand	MK764275	MK764341	MK764319	[41]
*N. petila*	MFLUCC 17-1738^T^	*Rhizophora mucronata*	Thailand	MK764276	MK764342	MK764320	[41]
*N. phangngaensis*	MFLUCC 18-0119^T^	*Pandanus* sp.	Thailand	MH388354	MH412721	MH388390	[46]
*N. piceana*	CBS 254.32	*Cocos nucifera*	Indonesia	KM199372	KM199452	KM199529	[17]
*N. piceana*	CBS 394.48^T^	*Picea* sp.	The UK	KM199368	KM199453	KM199527	[17]
*N. photiniae*	MFLUCC 22-0129^T^	*Photinia serratifolia*	China	OP498008	OP752131	OP753368	[18]
*N. protearum*	CBS 114178^T^	*Leucospermum cuneiforme*	Zimbabwe	JN712498	KM199463	LT853201	[61]
*N. psidii*	FMBCC 11.2^T^	*Psidium guajava*	Pakistan	MF783082	MH477870	MH460874	[52]
*N. rhapidis*	GUCC 21501^T^	*Rhododendron simsii*	China	MW931620	MW980441	MW980442	[34]
*N. rhizophorae*	MFLUCC 17-1551^T^	*Rhizophora mucronata*	Thailand	MK764277	MK764343	MK764321	[41]
*N. rhizophorae*	MFLUCC 17 1550	*Rhizophora mucronata*	Thailand	MK764278	MK764344	MK764322	[41]
*N. rhododendri*	GUCC 21504^T^	*Rhododendron simsii*	China	MW979577	MW980443	MW980444	[34]
*N. rhododendricola*	KUN-HKAS-123204^T^	*Rhododendron* sp.	China	OK283069	OK274147	OK274148	[62]
*N. rosae*	CBS 101057^T^	*Rosa* sp.	New Zealand	KM199359	KM199429	KM199523	[17]
*N. rosicola*	CFCC 51992^T^	*Rosa chinensis*	China	KY885239	KY885245	KY885243	[63]
*N. rosicola*	CFCC 51993	*Rosa chinensis*	China	KY885240	KY885246	KY885244	[63]
***N. rosicola***	**NM47-0706**	***Alpinia oxyphylla***	**China**	**PP621769**	**PP767806**	**PP767842**	**In this study**
*N. samarangensis*	CBS 115451	Unidentified tree	China	KM199365	KM199447	KM199556	[17]
*N. saprophytica*	MFLUCC 12-0282^T^	*Magnolia* sp.	China	JX398982	JX399017	JX399048	[17]
*N. scalabiensis*	CAA 1029^T^	*Vaccinium corymbosum*	Portugal	MW969748	MW934611	MW959100	[64]
*N. sichuanensis*	CFCC 54338^T^	*Castanea mollissima*	China	MW166231	MW218524	MW199750	[44]
*N. siciliana*	AC46	*Persea americana*	Italy	ON117813	ON209162	ON107273	[65]
*N. sonneratae*	MFLUCC 17-1744	*Sonneronata alba*	Thailand	MK764279	MK764345	MK764323	[41]
*N. sonneratae*	MFLUCC 17-1745^T^	*Sonneronata alba*	Thailand	MK764280	MK764346	MK764324	[41]
*N. steyaertii*	IMI 192475^T^	*Eucalyptus viminalis*	Australia	KF582796	KF582794	KF582792	[17]
*N. surinamensis*	CBS 450.74^T^	Soil under *Elaeis guineensis*	Suriname	KM199351	KM199465	KM199518	[17]
*N. subepidermalis*	CFCC 55160	*Rosa chinensis*	China	OK560699	OM117690	OM622425	[48]
*N. suphanburiensis*	MFLUCC 22-0126^T^	Unknown	Thailand	OP497994	OP752135	OP753372	[18]
*N. terricola*	HKAS 123213	*Paeonia suffruticosa*	China	OP082294	OP235982	OP204796	[31]
*N. thailandica*	MFLUCC 17-1730^T^	*Rhizophora mucronata*	Thailand	MK764281	MK764347	MK764325	[41]
*N. thailandica*	MFLUCC 17-1731	*Rhizophora mucronata*	Thailand	MK764282	MK764348	MK764326	[41]
*N. umbrinospora*	MFLUCC 12-0285^T^	Unidentified plant	China	JX398984	JX399019	JX399050	[16]
*N. vaccinii*	CAA 1059^T^	*Vaccinium corymbosum*	Portugal	MW969747	MW934610	MW959099	[64]
***N. vaccinii***	**JR31-0709**	***Alpinia oxyphylla***	**China**	**PP621736**	**PP767792**	**PP767828**	**In this study**
***N. vaccinii***	**JR41-0709**	***Alpinia oxyphylla***	**China**	**PP621738**	**PP767793**	**PP767829**	**In this study**
***N. vaccinii***	**JR44-0709**	***Alpinia oxyphylla***	**China**	**PP621739**	**PP767794**	**PP767830**	**In this study**
***N. vaccinii***	**JR51-0709**	***Alpinia oxyphylla***	**China**	**PP621740**	**PP767795**	**PP767831**	**In this study**
*N. vacciniicola*	CAA 1055^T^	*Vaccinium corymbosum*	Portugal	MW969751	MW934614	MW959103	[64]
*N. vheenae*	BRIP 72293a^T^	*Macadamia integrifolia*	Australia	MZ303792	MZ312685	MZ344177	[22]
*N. vitis*	MFLUCC 15-1265^T^	*Vitis vinifera* cv. “Summer black”	China	KU140694	KU140685	KU140676	[66]
*N. vitis*	MFLUCC 15-1270	*Vitis vinifera* cv. “Kyoho”	China	KU140699	KU140690	KU140681	[66]
***N. wuzhishanensis***	**YX116-0708^T^**	***Alpinia oxyphylla***	**China**	**PP621762**	**PP767818**	**PP767854**	**In this study**
*N. xishuangbannaensis*	KUMCC 21-0424^T^	*Kerivoula hardwickii* (bat)	China	ON426865	OR025934	OR025973	[67]
*N. xishuangbannaensis*	KUMCC 21-0425	*Kerivoula hardwickii* (bat)	China	ON426866	OR025935	OR025974	[67]
***N. yongxunensis***	**YX101-0708^T^**	***Alpinia oxyphylla***	**China**	**PP621760**	**PP767816**	**PP767852**	**In this study**
*N. zakeelii*	BRIP 72282a^T^	*Macadamia integrifolia*	Australia	MZ303789	MZ312682	MZ344174	[22]
*N. zimbabwana*	CBS 111495^T^	*Leucospermum cunciforme*	Zimbabwe	NA	KM199456	KM199545	[17]
*N. zingiber* *is*	GUCC 21001^T^	*Zingiber officinale*	China	MW930715	MZ683390	MZ683389	[30]
*Neopestalotiopsis* sp.2	CFCC 54340	*Castanea mollissima*	China	MW166235	MW218528	MW199754	[44]
*Neopestalotiopsis* sp.2	ZX22B	*Castanea mollissima*	China	MW166236	MW218529	MW199755	[44]
*Neopestalotiopsis* sp. nov.	GUCC 210003	Unknown	China	MW930717	MZ683392	MZ540914	[34]
*Neopestalotiopsis* sp.1	CFCC 54337	*Castanea mollissima*	China	MW166233	MW218526	MW199752	[44]
*Neopestalotiopsis* sp.1	ZX121	*Castanea mollissima*	China	MW166234	MW218527	MW199753	[44]
***Neopestalotiopsis*** **sp.3**	**SX11-0706**	***Alpinia oxyphylla***	**China**	**PP621748**	**PP767808**	**PP767844**	**In this study**
***Neopestalotiopsis*** **sp.4**	**MH133-0708**	***Alpinia oxyphylla***	**China**	**PP621753**	**PP767801**	**PP767837**	**In this study**
***Neopestalotiopsis*** **sp.5**	**XC11-0709**	***Alpinia oxyphylla***	**China**	**PP621747**	**PP767812**	**PP767848**	**In this study**
*Pestalotiopsis adusta*	ICMP 6088^T^	Refrigerator door	Fiji	JX399006	JX399037	JX399070	[16]
*P. adusta*	MFLUCC 10-0146	*Syzygium* sp.	Thailand	JX399007	JX399038	JX399071	[16]
*P. aggestorum*	LC6301^T^	*Camellia sinensis*	China	KX895015	KX895348	KX895234	[68]
*P. appendiculata*	CGMCC 3.23550^T^	*Rhododendron* sp.	China	OP082431	OP185516	OP185509	[69]
*P. australasiae*	CBS 114126^T^	*Knightia* sp.	New Zealand	KM199297	KM199409	KM199499	[17]
*P. australasiae*	CBS 11141	*Protea* sp.	New South Wales	KM199298	KM199410	KM199501	[17]
*P. australis*	CBS 114193^T^	*Grevillea* sp.	New South Wales	KM199332	KM199383	KM199475	[17]
*P. biciliata*	CBS 124463^T^	*Platanus* × *hispanica*	Slovakia	KM199308	KM199399	KM199505	[17]
*P. brachiata*	LC2988^T^	*Camellia* sp.	China	KX894933	KX895265	KX895150	[68]
*P. brassicae*	CBS 170.26^T^	*Brassica napus*	New Zealand	KM199379	NA	KM199558	[17]
*P. camelliae*	MFLUCC 12-0277^T^	*Camellia japonica*	China	JX399010	JX399041	JX399074	[16]
*P. camelliae-oleiferae*	CSUFTCC08^T^	*Camellia oleifera*	China	OK493593	OK562368	OK507963	[19]
*P. chamaeropis*	CBS 186.71^T^	*Chamaerops humilis*	Italy	KM199326	KM199391	KM199473	[17]
*P. chiangmaiensis*	MFLUCC 22-0127^T^	*Phyllostachys edulis*	Thailand	OP497990	OP752137	OP753374	[18]
*P. chiaroscuro*	BRIP 72970	*Sporobolus natalensis*	Australia	OK422510	OK423752	OK423753	[70]
*P. clavata*	MFLUCC 12-0268^T^	*Buxus* sp.	China	JX398990	JX399025	JX399056	[16]
*P. colombiensis*	CBS 118553^T^	*Eucalyptus eurograndis*	Colombia	KM199307	KM199421	KM199488	[17]
*P. daliensis*	CGMCC 3.23548^T^	*Rhododendron* sp.	China	OP082429	OP185518	OP185511	[69]
*P. diploclisiae*	CBS 115587^T^	*Diploclisia glaucescens*	China	KM199320	KM199419	KM199486	[17]
*P. diversiseta*	MFLUCC 12-0287^T^	*Rhododendron* sp.	China	NR 120187	JX399040	JX399073	[16]
*P. dracaenae*	HGUP4037^T^	*Dracaena fragrans*	China	NA	MT598645	MT598644	[71]
*P. dracaenicola*	MFLUCC 18-0913^T^	*Dracaena* sp.	Thailand	MN962731	MN962733	MN962732	[72]
*P. dracontomelon*	MFUCC 10-0149^T^	*Dracontomelon dao*	Thailand	KP781877	NA	KP781880	[73]
*P. endophytica*	MFLUCC 18-0932	Endophytic on healthy leaves of *Magnolia candoll*	Thailand	NR 172439	NA	MW417119	[74]
*P. ericacearum*	IFRDCC 2439^T^	*Rhododendron delavayi*	China	KC537807	KC537821	KC537814	[75]
*P. etonensis*	BRI P 66615	*Sporobolus jacquemontii*	Australia	MK966339	MK977634	MK977635	[58]
*P. formosana*	NTUCC 17-009^T^	Dead grass	China	MH809381	MH809385	MH809389	[63]
*P. furcata*	MFLUCC 12-0054^T^	*Camellia sinensis*	Thailand	JQ683724	JQ683708	JQ683740	[76]
*P. fusoidea*	CGMCC 3.23545^T^	Endophytic in fresh *Rhododendron delavayi* leaves	China	OP082427	OP185519	OP185512	[69]
*P. grevilleae*	CBS 114127^T^	*Grevillea* sp.	Australia	KM199300	KM199407	KM199504	[17]
*P. hawaiiensis*	CBS 114491^T^	*Leucospermum* sp.	Hawaii	KM199339	KM199428	KM199514	[17]
*P. hispanica*	CBS 115391^T^	*Protea ‘Susara’*	Spain	MH553981	MH554640	MH554399	[59]
*P. hydei*	MFLUCC 20-0135^T^	*Litsea Petiolata*	Thailand	MW266063	MW251112	MW251113	[55]
*P. hydei*	GUCC 21-0816	Dead twigs	China	OP753660	OP765909	OP753383	[18]
***P. hydei***	**BA11-0708**	***Alpinia oxyphylla***	**China**	**PP621755**	**PP767822**	**PP767858**	**In this study**
***P. hydei***	**BA42-0708**	***Alpinia oxyphylla***	**China**	**PP621756**	**PP767823**	**PP767859**	**In this study**
*P. hollandica*	CBS 265.33^T^	*Sciadopitys verticillata*	The Netherlands	KM199328	KM199388	KM199481	[17]
*P. humus*	CBS 336.97^T^	Soil	Papua New Guinea	KM199317	KM199420	KM199484	[17]
*P. hunanensis*	CSUFTCC15^T^	*Camellia oleifera*	China	OK493599	OK562374	OK507969	[19]
*P. iberica*	CAA1006^T^	*Pinus radiata*	Spain	MW732249	MW759036	MW759039	[77]
*P. inflexa*	MFLUCC 12-0270^T^	Unidentified tree	China	JX399008	JX399039	JX399072	[16]
*P. intermedia*	MFLUCC 12-0259^T^	Unidentified tree	China	JX398993	JX399028	JX399059	[16]
*P. jiangxiensis*	LC4399^T^	*Eurya* sp.	China	KX895009	KX895341	KX895227	[68]
*P. jinchanghensis*	LC6636^T^	*Camellia sinensis*	China	KX895028	KX895361	KX895247	[68]
*P. kandelicola*	NCYU 19-0355^T^	*Kandelia candel*	China	MT560722	MT563099	MT563101	[78]
*P. kaki*	KNU-PT-1804^T^	*Diospyros kaki*	Korea	LC552953	LC552954	LC553555	[79]
*P. kenyana*	CBS 442.67^T^	*Coffea* sp.	Kenya	KM199302	KM199395	KM199502	[17]
*P. kenyana*	CBS 911.96	Raw material from agar–agar	NA	KM199303	KM199396	KM199503	[17]
*P. knightiae*	CBS 114138^T^	*Knightia* sp.	New Zealand	KM199310	KM199408	KM199497	[17]
*P. knightiae*	CBS 111963	*Knightia* sp.	New Zealand	KM199311	KM199406	KM199495	[17]
*P. linearis*	MFLUCC 12-0271	*Trachelospermum* sp.	China	JX398992	JX399027	JX399058	[16]
*P. loeiana*	MFLUCC 22-0123	Dead leaves	Thailand	OP497988	OP713769	OP737881	[18]
*P. lushanensis*	LC4344^T^	*Camellia* sp.	China	KX895005	KX895337	KX895223	[68]
*P. macadamiae*	BRIP 63738B^T^	*Macadamia integrifolia*	Australia	KX186588	KX186680	KX186621	[56]
*P. malayana*	CBS 102220^T^	*Macaranga triloba*	Malaysia	KM199306	KM199411	KM199482	[17]
*P. monochaeta*	CBS 144.97^T^	*Quercus robur*	The Netherlands	KM199327	KM199386	KM199479	[17]
*P. jesteri*	MFLUCC 12-0279^T^	*Fagraea bodenii*	China	JX399012	JX399043	JX399076	[16]
*P. nanjingensis*	CSUFTCC16^T^	*Camellia oleifera*	China	OK493602	OK562377	OK507972	[19]
*P. nanningensis*	CSUFTCC10^T^	*Camellia oleifera*	China	OK493596	OK562371	OK507966	[19]
*P. neolitseae*	NTUCC 17-011^T^	*Neolitsea villosa*	China, Taiwan	MH809383	MH809387	MH809391	[63]
*P. oryzae*	CBS 353.69^T^	*Oryza sativa*	Denmark	KM199299	KM199398	KM199496	[17]
*P. papuana*	CBS 331.96^T^	Coastal soil	Papua New Guinea	KM199321	KM199413	KM199491	[17]
*P. photinicola*	GZCC 16-0028^T^	*Photinia serrulata*	China	KY092404	KY047663	KY047662	[80]
*P. rhizophorae*	MFLUCC 17-0416^T^	*Rhizophora apiculata*	Thailand	MK764283	MK764349	MK764327	[41]
*P. rhodomyrtus*	HGUP4230^T^	*Rhodomyrtus tomentosa*	China	KF412648	KF412642	KF412645	[47]
*P. rosarioides*	CGMCC 3.23549^T^	*Rhododendron decorum*	China	OP082430	OP185520	OP185513	[69]
*P. rosea*	MFLUCC 12-0258^T^	*Pinus* sp.	China	JX399005	JX399036	JX399069	[16]
*P. scoparia*	CBS176.25^T^	*Chamaecyparis* sp.	NA	KM199330	KM199393	KM199478	[17]
*P. shandogensis*	JZB340038^T^	Unknown	China	MN625275	MN626729	MN626740	[81]
*P. smilacicola*	MFLUCC 22-0125^T^	*Smilax* sp.	Thailand	OP497991	OP762673	OP753376	[18]
*P. suae*	CGMCC 3.23546^T^	*Rhododendron delavayi*	China	OP082428	OP185521	OP185514	[69]
*P. telopeae*	CBS 114161^T^	*Telopea* sp.	Australia	KM199296	KM199403	KM199500	[17]
*P. telopeae*	CBS 114137	*Protea* sp.	Australia	KM199301	KM199469	KM199559	[17]
*P. thailandica*	MFLUCC 17-1616^T^	*Rhizophora apiculata*	Thailand	MK764285	MK764351	MK764329	[41]
*P. trachycarpicola*	OP068^T^	*Trachycarpus fortunei*	China	JQ845947	JQ845945	JQ845946	[82]
*P. unicolor*	MFLUCC 12-0276^T^	*Rhododendron* sp.	China	JX398999	JX399030	NA	[16]
*P. verruculosa*	MFLUCC 12-0274^T^	*Rhododendron* sp.	China	JX398996	NA	JX399061	[16]
*P. yanglingensis*	LC4553^T^	*Camellia sinensis*	China	KX895012	KX895345	KX895231	[83]
*Pseudopestalotiopsis ampullacea*	LC6618^T^	*Camellia sinensis*	China	KX895025	KX895358	KX895244	[68]
*Ps. annellata*	NTUCC 17-030^T^	*Camellia sinensis*	China, Taiwan	MT322087	MT321889	MT321988	[23]
*Ps. avicenniae*	MFLUCC 17-0434^T^	*Avicennia marina*	Thailand	MK764287	MK764353	MK764331	[41]
***Ps. avicenniae***	**LF48-0709**	***Alpinia oxyphylla***	**China**	**PP621744**	**PP767825**	**PP767861**	**In this study**
*Ps. camelliae*	CGMCC 3.9192	*Camellia sinensis*	China	NA	KU562851	KU562850	[84]
*Ps. camelliae-sinensis*	NTUCC 18-031	*Camellia sinensis*	China, Taiwan	MT322047	MT321849	MT321948	[23]
*Ps. camelliae-sinensis*	LC3490^T^	*Camellia sinensis*	China	KX894985	KX895316	KX895202	[68]
*Ps. chinensis*	NTUCC 18-066	*Camellia sinensis*	China, Taiwan	MT322083	MT321885	MT321984	[23]
*Ps. chinensis*	LC3011^T^	*Camellia sinensis*	China	KX894937	KX895269	KX895154	[68]
*Ps. chinensis*	NTUCC 18-038	*Camellia sinensis*	China, Taiwan	MT322055	MT321857	MT321956	[23]
*Ps. cocos*	CBS 272.29^T^	*Cocos nucifera*	Java	MH855069	KM199467	KM199553	[17]
*Ps. celtidis*	GUCC 21599^T^	*Celtis sinensis*	China	OL423535	OL439010	OL439012	[33]
*Ps. curvatispora*	MFLUCC 17-1723	*Rhizophora mucronata*	Thailand	MK764290	MK764356	MK764334	[41]
*Ps. curvatispora*	MFLUCC 17-1722^T^	*Rhizophora mucronata*	Thailand	MK764289	MK764355	MK764333	[41]
*Ps. dawaina*	INPA 2912	*Caryota mitis*	Brazil	MN096659	MN151310	MN151308	[85]
*Ps. dawaina*	MM14-F0015^T^	Unknown	Dawei, Myanmar	LC324750	LC324751	LC324752	[86]
*Ps. gilvanii*	INPA 2913^T^	*Paullinia cupana*	Brazil	MN385951	MN385954	MN385957	[29]
*Ps. hydeae*	NTUCC 17-003.1	*Diospyros* sp.	China, Taiwan	MG816313	MG816323	MG816333	[87]
*Ps. ignota*	NN 42909^T^	*Camellia sinensis*	China	KU500020	NA	KU500016	[84]
*Ps. indica*	CBS 459.78^T^	*Hibiscus rosa-sinensis*	India	KM199381	KM199470	KM199560	[17]
*Ps. indocalami*	GUCC 21600^T^	*Indocalamus tessellatus*	China	OL423536	OL439011	OL439013	[33]
*Ps. ixorae*	NTUCC 17-001.1^T^	*Lxora* sp.	NA	MG816316	MG816326	MG816336	[87]
*Ps. kawthaungina*	MM14F0083^T^	Unknown	Kawthaung, Myanmar	LC324753	LC324754	LC324755	[86]
*Ps. kubahensis*	UMAS-KUB-P20^T^	*Macaranga* sp.	Sarawak, Malaysia	MG818971	NA	NA	[88]
*Ps. myanmarina*	NBRC 112264^T^	*Averrhoa carambola*	Dawei, Myanmar	LC114025	LC114045	LC114065	[89]
***Ps. myanmarina***	**JR34-0709**	***Alpinia oxyphylla***	**China**	**PP621737**	**PP767824**	**PP767860**	**In this study**
*Ps. rhizophorae*	MFLUCC 17-1560^T^	*Rhizophora apiculata*	Thailand	MK764291	MK764357	MK764335	[41]
*Ps. simitheae*	KUMCC 17-0255	*Magnolia candolli*	China	MW244023	MW602387	MW273930	[74]
*Ps. simitheae*	MFLUCC12-0121^T^	*Pandanus odoratissimus*	Thailand	KJ503812	KJ503815	KJ503818	[90]
*Ps. solicola*	CBS 386.97^T^	Soil in tropical forest	Papua New Guinea	MH554039	MH554715	MH554474	[59]
*Ps. taiwanensis*	NTUCC 17-002.1^T^	*Ixora* sp.	China, Taiwan	MG816319	MG816329	MG816339	[87]
*Ps. thailandica*	MFLUCC 17-1724^T^	*Rhizophora mucronata*	Thailand	MK764292	MK764358	MK764336	[41]
*Ps. thailandica*	MFLUCC 17-1725	*Rhizophora mucronata*	Thailand	MK764293	MK764359	MK764337	[41]
*Ps. theae*	MFLUCC 12-0055^T^	*Camellia sinensis*	Thailand	JQ683727	JQ683711	JQ683743	[16]
*Ps. vietnamensis*	NBRC 112252	*Fragaria* sp.	Hue, Vietnam	LC114034	LC114054	LC114074	[89]

Ex-type strains are labeled with ^T^. NA: not available. The strains in this study are indicated in bold font.

## Data Availability

All sequence data are available in NCBI GenBank following the accession numbers in the manuscript.
